# Supplementary material for: Experts’ views on how to design a tobacco control fund in the UK
Source: BMJ Open. 2022 Nov 28;12(11):e066224. doi: 10.1136/bmjopen-2022-066224 (PMC9710323; doi:10.1136/bmjopen-2022-066224)
Supplement: Supplementary data [file bmjopen-2022-066224supp001.pdf]

Appendix A: Interview topic guide

Part 1: Introduction/background

- First of all, thank you for taking part in this research.
- I'm [researcher name], a research assistant at the University of Glasgow with an interest in the communication of health issues and policies.
- Your will be aware from the participant information sheet that this is a project funded by Cancer Research UK to collect expert views on the extent to which a direct levy on the tobacco industry (a so called 'polluter pays' levy) might be an effective tobacco control measure. We are looking to compare the different forms of a levy and their potential impacts; consider how funds generated could be used for tobacco control activities; learn from international case studies; and if there is support for the levy, to provide recommendations for the next steps in advocating for it. Cancer Research UK (CRUK) will use the outcome of this project to inform their future tobacco control policy strategy.
- We are interviewing a mix of stakeholders with relevant expertise including legal experts, economists, financial or tax advisors, industry experts or representative bodies, charities and harm-reduction groups and academics or researchers.

Key points for consent:

- Can I confirm that you have received the participant information sheet and signed and returned your consent form.
- Just to reiterate, your taking part is voluntary, and you are free to withdraw at any time without giving a reason, up until the point where the data is published in an NHS Health Scotland report.
- Your participation will be anonymous. Excerpts from the interview may be quoted verbatim in a report and a paper, but quotations will be anonymised to avoid accidentally disclosing your identity. We realise that within certain policy communities it may be possible for others to identify you from your experiences of specific policies, as such we will take care to anonymise quotations as appropriate to avoid accidental disclosure. Non-anonymised interview recordings and transcripts will be destroyed securely upon completion of this research, but anonymised transcripts and consent forms will be stored securely by the University of Glasgow for a period of 10 years for the purposes of ensuring research integrity.
- Finally, the study has been approved by the Ethics Committee of the College of Social Sciences at the University of Glasgow.

Verbal confirmation of consent:

- Do you consent to take part in this research, and do you give consent for me to record this interview?
- Can you describe your professional role, and how it relates to taxation, tobacco control or the regulation of unhealthy commodities?

Part 2: How to raise funds? [Note: can focus on this more or less depending on interviewee's areas of expertise]

- What different mechanisms are you aware of for raising funds from the tobacco industry?
    - o How about hypothecated excise taxes? [explain if necessary]

            - Do you think excise taxes are an effective away to raise revenue?
                - What are the pros and cons?
                - Could that revenue be effectively ring-fenced for specific purposes?
            - What specifically should a tax be applied to?
                - Sales at retail
                - Sales between manufacturers and distributors, or between distributors and retailers?
                - Sales crossing international boundaries
            - [If participant is favourable towards excise taxes]
                - Should tax rates differ between different tobacco products, such as heat not burn products, or should there be a flat rate of tax across all tobacco products?
                - Should taxes be direct (fixed amount per unit) or ad valorem (percentage of price)?
        - How about an industry levy? [explain if necessary – mandatory direct tax charged to the industry]
            - Are you aware of any direct taxes or levies that already exist?
                - Prompt: Soft drinks industry levy
                - Prompt: Pharmaceutical Price Regulation Scheme (UK)
                - Prompt: Tobacco product user fees (US)
        - Are there any examples of where other countries have used taxes or levies to raise funds from the tobacco industry for tobacco control? What lessons might we take from these?
        - Are there any novel approaches that should be considered?
            - Are there useful lessons to be learned from other pricing and taxation schemes (ECO, MUP, SDIL, single-use bag tax, Scottish Landfill Communities Fund)
- How far in advance should a fundraising scheme be planned?
    - Should funding rise over time, remain steady or drop over time?
    - At what point will it no longer be necessary?
    - What time of ongoing surveillance of the market will be necessary?
- What ways might the tobacco industry try to avoid contributing more? What are the implications of these strategies? How might we prevent these?
    - Prompt: TTCs 'hiding' profits in other countries
    - Prompt: over-shifting (increase prices on top of tax increases)
    - Prompt: under-shifting (absorb tax increases to keep retail prices stable
    - Prompt: brand-shifting (over- and under-shift different brands to encourage continued use and initiation of tobacco
    - Prompt: Collusion between TTCs to keep prices low
    - Prompt: Counter-marketing to undermine investment in public communication campaigns
    - Prompt: Subversion of a tobacco control fund idea eg: PMI's Tobacco Transition Fund
    - Prompt: Use of a TTF to gain access to policy making
    - Anything else?
- Is raising the price of tobacco products desirable? How does it relate to inequalities?
    - [If undesirable] Is capping prices a good idea? Is it practically and legally feasible?
    - Can you think of any wider economic impacts of extracting more revenue from the tobacco industry or tobacco trade?
- Who should administer fundraising? An existing system or organisation? A newly-formed organisation?
- How might we determine what is reasonable and affordable for tobacco companies to contribute?
    - How about the profits they make, relative to other commodities?

        - Prompt: the tobacco industry are one of the most profitable businesses in the world and make over £1bn in profit in the UK per year. Tobacco businesses tend to enjoy profit margins of up to 68%, compared to 15-20% in most staple consumer industries.
        - Prompt: however, tobacco sales have declined with the covid-19 pandemic
    - How about the amounts that they used to spend on advertising, before tobacco advertising was prohibited?
        - Prompt: evidence suggests that they used to spend £144m a year on advertising in the UK, adjusted for inflation
    - How about setting a target amount to raise in order to fund effective tobacco control activities at local, regional and national level, and then apportioning that across the tobacco industry?
    - How to apportion contributions between different companies?
        - Just the big four transnational tobacco companies, or any manufacturer or importer of tobacco operating in the UK?
        - Apportion by each company's share of the combustible tobacco product market? Or other products? Historical data vs current data?
- Should the tobacco industry be incentivised to move out of the combustible tobacco market, and encourage smokers to transition to alternative nicotine containing products?

<u>Part 3: How to disburse funds?</u> [Note: can focus on this more or less depending on interviewee's areas of expertise]

- What are the pros and cons of ring-fencing raised funds for a specific purpose vs. adding those funds to general public revenue?
- What should funds be used for?
    - Tobacco control measures
        - Cessation services
        - Preventing young people from initiating smoking
        - Fighting illicit tobacco
        - Mass media, social marketing and educational campaigns
        - Enforcement for age of sale compliance
        - Environment, parks and recreational resources
        - Any other ideas?
        - Is addition funding for tobacco control necessary if we are already trending towards tobacco being effectively obsolete?
    - General health costs or health promotion (not limited to tobacco)
    - Should the uses of funds be determined centrally or locally (i.e., by each devolved administration, or by regions/authorities within the devolved nations?)
    - Should the use of funds be set in stone, or flexible? How to make sure changes in the use of funds are sensible?
    - Is there a way to make sure funding addresses health inequalities?
- How to ensure that funds are disbursed for the intended purposes?
- Who should oversee and regulate the disbursement of funds, and why?
    - DHSC and their equivalents in the devolved nations?
    - A newly-formed semi-independent body?
    - A committee of appointed experts from government and civil society?

<u>Part 4: Advocacy, communication and legislation</u>

Hilton S, *et al. BMJ Open* 2022; 12:e066224. doi: 10.1136/bmjopen-2022-066224

- How are the tobacco industry likely to respond to the announcement of a tax or levy?
    - Prompt: the industry has a track record of interfering with policy development and implementation
    - How to respond to likely industry critiques?
        - Example: tax is unfair and regressive
        - Example: revenue will not be used effectively
        - Example: taxes will be passed on to consumers
        - Example: higher costs will encourage illicit trade
        - Example: will harm the economy and endanger jobs
        - Example: revenue will decrease as purchasing decreases
        - Example: we're already working to transition people to reduced risk products therefore no further regulation necessary (CSR arguments)
- What other challenges do you anticipate in advocating for a new tax or levy?
    - Prompt: lobbying, media campaign, CSR rhetoric, reduced risk products, legal challenges
- What opportunities are there for advocating for a new tax or levy?
    - Prompt: Evidence of public support for raising taxes to pay for health and tobacco control
    - Prompt: Support from All Party group on Smoking and Health and the Smokefree Action Coalition
    - Prompt: money invested in tobacco control tends to create a large return on investment through healthcare savings
      Prompt: WHO FCTC requires that the government stringently regulates the tobacco market
- Is 'polluter pays' a useful way of framing a tobacco industry levy to fund tobacco control?
- What type of legislation is likely to be necessary to implement the types of measures we have discussed?
    - How long could we expect it to take

<u>Part 5: Contextual factors</u>

- How do you think the devolved nature of the UK will affect a new tax or levy?
    - How about giving devolved nations the option to opt into a scheme originating in Westminster? If so, to what extent should they have autonomy over how funds are used
    - How to calculate the distribution of funds between each of the devolved nations involved in the scheme?
- Do you have a feeling for how Brexit might affect a new tax or levy?
    - Are any challenges or opportunities presented by the change in the legal context caused by leaving the EU?
- How might Covid-19 affect a new tax or levy?
    - Prompt: tobacco sales have dropped during the pandemic
    - Prompt: covid-19 is expected to cause a global recession, how might that influence things?
    - Prompt: Would a tobacco control levy be an acceptable way of raising funds for economic recovery?
    - Prompt: Will Covid-19 raise the profile of NCD prevention measures such as tobacco control?
    - Prompt: Might the pandemic affect public and political attitudes to NHS funding? How might it change how we view policy solutions to NCDs and unhealthy commodities?
    - Prompt: How can we keep the issue of tobacco control on the political agenda?

Close interview

- Is there anything else you would like to add, that we haven't already talked about?
- Thank you very much for taking part.
- If appropriate – ask if they are interested in taking part in the second phase discussion groups.

Hilton S, *et al. BMJ Open* 2022; 12:e066224. doi: 10.1136/bmjopen-2022-066224
