# Supplementary material for: Experts’ views on how to design a tobacco control fund in the UK
Source: BMJ Open. 2022 Nov 28;12(11):e066224. doi: 10.1136/bmjopen-2022-066224 (PMC9710323; doi:10.1136/bmjopen-2022-066224)
Supplement: Author's manuscript [file bmjopen-2022-066224.draft_revisions.pdf]

BMJ Open

BMJ Open is committed to open peer review. As part of this commitment we make the peer review history of every article we publish publicly available.

When an article is published we post the peer reviewers' comments and the authors' responses online. We also post the versions of the paper that were used during peer review. These are the versions that the peer review comments apply to.

The versions of the paper that follow are the versions that were submitted during the peer review process. They are not the versions of record or the final published versions. They should not be cited or distributed as the published version of this manuscript.

BMJ Open is an open access journal and the full, final, typeset and author-corrected version of record of the manuscript is available on our site with no access controls, subscription charges or pay-per-view fees (http://bmjopen.bmj.com).

If you have any questions on BMJ Open's open peer review process please email info.bmjopen@bmj.com

BMJ Open

How to fund a generation free from tobacco in the UK? Expert views on designing a Tobacco Control Fund

Journal:	*BMJ Open*
Manuscript ID	bmjopen-2022-066224
Article Type:	Original research
Date Submitted by the Author:	04-Jul-2022

Complete List of Authors:	Hilton , Shona; University of Glasgow MRC/CSO Social and Public Health Sciences Unit<br>Smith, Marissa; University of Glasgow MRC/CSO Social and Public Health Sciences Unit<br>Buckton, Christina; University of Glasgow MRC/CSO Social and Public Health Sciences Unit<br>Patterson, Chris; University of Glasgow MRC/CSO Social and Public Health Sciences Unit

Keywords:	PUBLIC HEALTH, QUALITATIVE RESEARCH, Health policy < HEALTH SERVICES ADMINISTRATION & MANAGEMENT

SCHOLARONE™
Manuscripts

I, the Submitting Author has the right to grant and does grant on behalf of all authors of the Work (as defined in the below author licence), an exclusive licence and/or a non-exclusive licence for contributions from authors who are: i) UK Crown employees; ii) where BMJ has agreed a CC-BY licence shall apply, and/or iii) in accordance with the terms applicable for US Federal Government officers or employees acting as part of their official duties; on a worldwide, perpetual, irrevocable, royalty-free basis to BMJ Publishing Group Ltd ("BMJ") its licensees and where the relevant Journal is co-owned by BMJ to the co-owners of the Journal, to publish the Work in this journal and any other BMJ products and to exploit all rights, as set out in our licence.

The Submitting Author accepts and understands that any supply made under these terms is made by BMJ to the Submitting Author unless you are acting as an employee on behalf of your employer or a postgraduate student of an affiliated institution which is paying any applicable article publishing charge ("APC") for Open Access articles. Where the Submitting Author wishes to make the Work available on an Open Access basis (and intends to pay the relevant APC), the terms of reuse of such Open Access shall be governed by a Creative Commons licence – details of these licences and which Creative Commons licence will apply to this Work are set out in our licence referred to above.

Other than as permitted in any relevant BMJ Author's Self Archiving Policies, I confirm this Work has not been accepted for publication elsewhere, is not being considered for publication elsewhere and does not duplicate material already published. I confirm all authors consent to publication of this Work and authorise the granting of this licence.

For peer review only

How to fund a generation free from tobacco in the UK? Expert views on designing a Tobacco Control Fund

Shona Hilton[1], Marissa J. Smith[1*] Christina Buckton[1] and Chris Patterson[1]

[1] MRC/CSO Social and Public Health Sciences Unit, University of Glasgow, Glasgow, UK

*Corresponding author

Email: Marissa.Smith@glasgow.ac.uk Tel: (+44) 0141 353 7500

Keywords: Public policy, Tobacco industry, Health services

Word count: 3500

For peer review only

Abstract

Objective

To explore expert views on the potential value, and approaches to establishing and administering a tobacco control fund in the United Kingdom (UK).

Design

Semi-structured interviews and follow-up discussion groups.

Subjects

Twenty-four UK and international experts on tobacco control regulation, public health, economics, or law from the academic, public, private and third sector.

Methods

Participants considered the relative merit of 1) general excise tax on retail tobacco sales; 2) ring-fenced hypothecation of excise taxes on retail tobacco sales; and 3) a direct levy on tobacco manufacturers. Preliminary synthesis of interview findings was deliberated upon in two follow-up discussion groups to identify key considerations for policy design.

Result

Most experts agreed that a ring-fenced tobacco control fund would be valuable method of raising predictable and reliable funds from tobacco producers either using either companies' sales volume or market share as a way to establish the proportion they should pay. Experts suggested the fund in the UK should be administered by a government body with devolved nation input and with an independent advisory group. They suggested it should be allocated yearly with a distribution at local, regional, and national levels to support smoking prevention and cessation rather than treatment activities with priority given to measures that tackle smoking-related inequalities.

Conclusion

For peer review only - http://bmjopen.bmj.com/site/about/guidelines.xhtml

There was overwhelming agreement by experts of the need to establish a tobacco control fund to help meet the proposed government tobacco free targets to reduce adult smoking prevalence to 5% by 2030 (UK) and 2034 (Scotland).

What is already known on this subject?

- Tobacco is highly addictive, and each year kills more than 8 million worldwide
- The economic costs of tobacco use continue to be greater than the costs for treating tobacco related diseases
- The UK and devolved nations smoking targets are not likely to be met unless new funding is made available

What this paper adds?

- Little research has been conducted with experts examining support for raising revenue for a tobacco control fund from the tobacco industry
- This paper offers early insights into how a tobacco control fund might be established and administered in the UK
- We identify a set of key foundational principles that must be engaged with in designing a tobacco control fund policy in the UK

For peer review only

Introduction

Worldwide tobacco kills more than 8 million people per annum [1] including nearly 100,000 preventable deaths in the UK [2]. Tobacco is highly addictive, there is no safe level of exposure and all forms of tobacco are harmful to health increasing the risk of cancers, heart disease and other NCDs [1]. Despite a broad range of effective tobacco control policies, the tobacco trade continues to be highly profitable [3]. In contrast, the economic costs of tobacco use in society are greater than the

For peer review only - http://bmjopen.bmj.com/site/about/guidelines.xhtml

costs for treating tobacco related diseases. For example in the UK, revenue from excise duty on tobacco sales continues to be substantially lower than health costs of smoking [4]. While UK smoking prevalence has declined precipitously in response to tobacco control action [5, 6], the smoking inequality gap has grown [6] as smoking contributes to poverty by diverting household spending from basic needs such as food and shelter to tobacco.

To further reduce smoking, the UK and Scottish governments have proposed targets to reduce adult smoking prevalence to 5% by 2030 and 2034, respectively [7, 8]. However, for these proposed targets to be met prevalence rates need to decline at a much faster rate [9] which may require additional policy to support tobacco control measures.

One policy under consideration is to establish a ring-fenced tobacco control fund. This system for health promotion has been pursued in other countries including Australia, Vietnam, Korea and Thailand [10-12]. In the UK in 2015, HM Treasury published their conclusions on an earlier consultation on the potential design of a levy on tobacco manufacturers and importers [13]. The consultation considered a tobacco levy under the administration of the existing corporation tax system, imposed on manufacturers and importers of products on which tobacco excise duty is paid [14]. Whilst the proposal received the support of a broad range of health charities, professional bodies and academics; the UK Government decided not to pursue the tobacco levy, citing concerns that costs would be passed on to consumers and that tobacco sales are already subject to escalating duties [14]. Since the government rejected this tobacco industry levy, other fiscal approaches to tackling the harms caused by unhealthy products have been introduced, including Scotland's Minimum Unit Pricing for alcohol [15], and the 'soft hypothecation' of the UK's Soft Drinks Industry Levy (SDIL) [16]. In light of an increasing political willingness to implement other fiscal interventions, and the continued advocacy for a tobacco control fund from the public health community [17, 18], raising revenue for tobacco control fund from the tobacco industry remains a viable policy option. This paper explores contemporary views of UK and international tobacco control and public health experts on the potential value of, and approaches to establishing and administering a tobacco control fund. In doing so we identify key considerations for its design.

For peer review only - http://bmjopen.bmj.com/site/about/guidelines.xhtml

Methods

Interviews

We developed a purposive sampling frame to target UK and international experts in tobacco control regulation, public health, economics, or law from the academic, public, private and third sector. Twenty-four experts agreed to participate after reading the participant information sheet, privacy notice and signing the consent form. Eighteen were based in the UK, four in the United States and two in South Africa. Table 1 illustrates the distribution of participants by the sector in which they primarily worked and their principal topic of work. Participants classified as 'public health' were those who work and publish with the broader area of public health. Participants classified as 'tobacco control' were those who had expertise in the area of tobacco control and predominately work and publish in the tobacco control area.

A semi-structured interview schedule was informed by reviewing international academic and grey literature on tobacco control funds. The interviews were conducted between September 2020 and January 2021. One interview was conducted by telephone and the remaining 23 interviews were conducted using Microsoft Teams video meetings. The interviews lasted between approximately 45 and 60 minutes, all were recorded and transcribed verbatim.

Table 1: Sample composition by primary sector of work and primary area of expertise

**Primary sector**	**Primary area of expertise**			**Total**
	Economics / Law	Public Health	Tobacco control	
Academia	6	3	0	**9**
Public sector	0	4	3	**7**
Third sector	1	1	5	**7**
Private sector	1	0	0	**1**

For peer review only - http://bmjopen.bmj.com/site/about/guidelines.xhtml

**Total**	**8**	**8**	**8**	**24**

Discussion Groups

In March 2021, two follow-up online discussion groups were conducted with nine individuals using Microsoft Teams. Participants were selected for these follow-ups based on their sectorial expertise and to represent key disciplines. The first discussion group included three third sector professionals with expertise spanning tobacco control and public health advocacy and two academic economists. The second discussion group included two public sector professionals with roles in tobacco control and public health policy, and two academics with expertise in law and public health. The aim of these groups was to consider the synthesis of views from the interviews on the potential value of a tobacco control fund and to identify key considerations for policy design. Each discussion group lasted two hours, and group discussions were recorded for later checking against the minutes.

Analysis

Data analysis consisted of interview transcriptions and discussion group minutes. After reading and re-reading the transcripts a coding frame was developed iteratively by the research team organising data into inductive categories. NVivo was used to organise categories on the basis of themes with similar features this allowed a systematic capture of both areas of agreement and less typical perspectives across a range of categories. The discussion groups recordings and minutes were cross compared with the interview coding frame to confirm and expand on codes relating to recommendations for policy design of a tobacco control fund. To maintain participant anonymity as agreed at consent, brackets have been used to replace identifiably details about professional activities.

Ethical approval for this study was granted on by the College of Social Sciences Research Ethics Committee at the University of Glasgow (reference 400190213).

For peer review only - http://bmjopen.bmj.com/site/about/guidelines.xhtml

Patient and Public Involvement

None.

Results

What is the potential value of a tobacco control fund?

There was general agreement that a tobacco control fund could be a valuable revenue for raising predictable and reliable funds direct from the tobacco industry. Typically, this was viewed as a way to boost current public health efforts: "*The more money that we can earmark, ring-fence into public health and tobacco control efforts the better from a public health point of view*" (P02, academic, law). However, two participants cautioned that whist such a fund was largely welcomed, it would be important that a tobacco control fund did not act as a disincentive for government funding or cutbacks to existing tobacco control activities. Participants also welcomed the fact that an industry-funded payment, would help hold the tobacco industry more accountable for the damage they cause to society, with one participant stating: "*There's some sort of nice symmetry about money from the tobacco industry being used to improve or solve some of the problems it creates*" (P05, third sector, public health). However, participants also warned that the funding mechanism of extracting money from the tobacco industry would need careful consideration so that levy was not passed on to nicotine-dependent and socially deprived smokers, with one participant warning that: "*It doesn't really make sense, I think, to pursue further interventions that actually further widen the health inequality that we have*" (P07, academic, public health).

How might a tobacco control fund be designed?

In thinking about general principles of where this fund might come from, participants discussed considered three options from 'profits', 'sales volume' or 'market share'. The option of a payment

For peer review only - http://bmjopen.bmj.com/site/about/guidelines.xhtml

coming directly from 'profits' was largely discounted on the grounds that 'multinational companies are very good at moving money around and shifting profits to other countries with lower tax systems' (P03, academic, public health). The option of using 'sales volume' or 'market share' were both more popular as they were deemed more difficult for companies to obscure and shift money. Examples given were: "*The harm is linked to the sales volume of the product, not to the profits they make from it" (P23, third sector, tobacco control).* Or that: "*Market share is the easiest way to do it. And you may want to average market share over the past 30 years or something like that to try to figure out what it is." (P02, academic, law)*

Participants then considered in more detail how a tobacco control fund might be designed to raise funds: 1) general excise tax on retail tobacco sales; 2) ring-fenced hypothecation of excise taxes on retail tobacco sales; and 3) a direct levy on tobacco manufacturers. Participants were asked to consider the relative merits of each funding approach.

General excise tax on retail tobacco sales

Participants were widely supportive of excise taxes, predominantly for their role in decreasing consumption, but also for their role in fundraising with some participants drawing on excise tax in Australia and New Zealand as useful models for the UK policymakers to consider. Participants highlighted the simplicity, efficiency, and political acceptability of excise tax as positive attributes of this approach. Some participants expressed doubt about the usefulness of excise taxes to fundraising given falling revenue with one academic stating:

> *"The UK I know is now sort of sitting at the top of that revenue situation where they increased excise tax, revenues are not increasing all that much because the excise taxes are very high already." (P01, academic, economics)*

In contrast , other participants suggested that the government can effectively control the extent to which taxes are passed on to consumers by capping retail prices, meaning that increasing specific excise tax can raise revenue while ensuring that retail prices do not increase so consumers do not bear the additional cost.

For peer review only - http://bmjopen.bmj.com/site/about/guidelines.xhtml

Ring-fenced hypothecated excise tax

Participants discussed the potential for some or all of excise taxation on retail tobacco sales to be hypothecated, meaning that it would be diverted into a specific fund instead of general government funds. This approach was viewed by participants as publicly acceptable as explained:

> *"Dealing with the consequences or addressing the harms that arise from the product I think is actually instinctively appealing to people." (P22, public sector, public health)*

However, while appealing in principle, participants overwhelmingly indicated that hypothecation would meet with too much opposition from HM Treasury, as noted by one academic: "*Politicians in general don't like it, they're very particular about being elected to do the right thing, and they wish to retain their independence and their freedom for manoeuvre. So, it can be a challenging negotiation that one"* (P03, academic, public health).

Despite this view, participants identified the UK's SDIL as a possible route to hypothecation but noted that the funds raised by SDIL were not ultimately ring-fenced for the purposes they originally presented to the public, with one participant noting:

> *"The sugar tax was pushed through with major public support on the basis of hypothecation. And then guess what? There was a crisis and the money, the revenues raised for the sugar tax miraculously didn't get spent on children's breakfast clubs and school sports but have been used to fill gaps in broader public health, and possibly NHS budgets as well. That's always a risk". (P03, academic, public health).*

This led to discussions about how to win political support for hypothecation and the merits of creating a general health fund instead of a tobacco control fund. As explained by one participant: "*I*

For peer review only - http://bmjopen.bmj.com/site/about/guidelines.xhtml

absolutely expect that it would be easier to convince policymakers, who generally don't like hypothecated taxes, [of the merits of a general health fund] so the more freedom that you give them, the more acceptable it's likely to be. But I would rather think it probably would be less acceptable to the public, because … if you're using the sort of, polluter pays type principle, then, you know, people expect that there is a direct consequence between those two things." *(P22, public sector, public health)*. Another potential route to hypothecation discussed that bypassed HM Treasury was: "*If it was seen as a user fee done by the Department of Health and Social Care, then it would bypass the treasury's normal functioning" (P09, academic, economics)*. Bypassing HM Treasury was advocated by three participants who suggested taking inspiration from the UK's Pharmaceutical Price Regulation Scheme (PPRS), through which the Department for Health and Social Care (DHSC) (and not the treasury) receives excess profits from participating pharmaceutical companies and uses that funding to address shortfalls in NHS budgets due to expenditure on novel treatments. While this scheme is not an example of hypothecated tax, it was presented as precedent for the DHSC receiving funds from industry, and an illustration of relevant administrative expertise within DHSC, as explained:

> *"The important thing from the tobacco point of view is, you've established this principle of soft hypothecation where the rebates from the industry go back specifically for…or back to the [DHSC], rather just the Treasury who just grab it." (P24, private sector, pharmaceuticals)*

A levy on the tobacco industry

Participants who favoured this approach typically viewed it as a means to extract funds from industry instead of from consumers, which may be more appealing to the public and could help convince policymakers:

> *"I think politically it's more sellable to the public [than excise tax increases]." (P08, third sector, tobacco control)*

For peer review only - http://bmjopen.bmj.com/site/about/guidelines.xhtml

> *"I think that would be a decision-making factor for any governmental policy measure that got put forward, that it would be very much clear that the industry would be the contributor, not the public, if you like." (P22, public sector, public health)*

Participants who favoured a levy typically viewed it as a means to extract funds from industry instead of from consumers, making it more appealing to the public and policymakers: "*I think politically it's more sellable to the public [than excise tax increases].*" (P08, third sector, tobacco control) "*It would be attractive that the industry would be the contributor, not the public."* (P22, public sector, public health). Controlling retail prices was deemed an essential part of ensuring that the cost of a levy is borne by the industry.

> *"The way that the tobacco companies are monopolies and making excess profits is because they are using gradual escalator duty increases to increase their own prices. So you need to cap prices" (P24, private sector, pharmaceuticals).*

Similarly, another participant suggested that limiting retail prices needed to be part of a levy and could have an added benefit of disincentivising tobacco companies' continued involvement in the UK market. This participant considered that: "*If tobacco had to be sold at the price it cost to make and distribute, you know, and box it, package, get it here, sell it and VAT paid and profit, it's already a pretty unprofitable product anyway and it's only the economies of scale that get it through."* (P16, public sector, tobacco control)

Conversely, some participants argued that retail prices should not be limited as price increases are beneficial in reducing consumption: As explained: "*When you do see tax increases, you tend to see over-shifting of the tax and using that as an opportunity to raise price and capitalise on at least the addicted consumers that are still in the market. So that is happening, but I don't know that that's necessarily a bad thing, because in the end those price increases are also very effective and leading to additional cessation and particularly in terms of preventing initiation." (P04, academic, economics)*

For peer review only

The PPRS was presented as a potential model for extracting industry profits outside of excise taxes, and one that has been refined over many years to limit potential loopholes. However, PPRS was generally considered to be of limited use in having real world transferability from pharmaceuticals to tobacco. One public sector participant stated that the "*UK pharmaceutical market's status as a virtual monopsony differs starkly from the tobacco industry and suggested that such a scheme may incentivise the lowering of tobacco prices" (P14, public sector, tobacco control).*

Other policy design considerations

After considering the different options for designing a tobacco control fund, participants considered other factors that would be essential for gaining public and political support. A key factor identified was the need for the fund to be administered by a government body with an independent advisory group to ensure transparent decision making. As highlighted by one participant suggesting a requirement would be: "*a transparent body that both industry and [academic] researchers and the government had trust in to operate transparently and fairly and not be unduly influenced by any stakeholders, you just need to make it an independent body.*" (P05, third sector, economist)

It was also agreed that the fund should be allocated yearly with a distribution at local, regional and national levels to support smoking prevention and cessation rather than treatment activities with priority given to tackling smoking-related inequalities in the most deprived areas. This was deemed important for: "*making smoking obsolete, to massively benefit the most deprived communities both economically as well as in health terms" (P18, public sector, public health).*

Discussion

Experts considered three broad approaches to raising funds: raising existing excise tax on tobacco sales, introducing a hypothecated excise tax and a tobacco industry levy Each approach was assessed as having strengths and weaknesses, for example, raising excise taxes was seen as politically feasible

For peer review only - http://bmjopen.bmj.com/site/about/guidelines.xhtml

and administratively simple, while hypothecation was seen as least politically plausible due to potential Treasury resistance and a tobacco levy was levy was deemed as a logical advocacy route following the polluter pays principle to ensure the industry pays for its damage to society. Experts agreed that whichever mechanism is chosen, this must be clearly guided by what the fund is directly trying to achieve. This is consistent with a recent Public Health England report on fiscal and pricing policies [19], which highlights that policy success depends on clarity of policy goals. Most experts agreed that key principles underlying the design of a fund would be to collect predictable and reliable funds from transnational tobacco producers either from companies' sales volume or their market share as a way to assign responsibility and establish the proportion they should pay. There was agreement that any fundraising mechanism which extracts funds from industry and avoids the potentially regressive effects of price increases on consumers may be the optimal fundraising approach. However, there was acknowledgement that policy goals have trade-offs. For example to achieve both health promotion and revenue raising objectives is possible within the same policy when demand for a product is relatively price inelastic, as is the case with tobacco [19]. From this perspective, permitting costs to be passed on to customers and ensuring that costs are paid by industry may each be valid goals and designing the policy requires skill.

In considering the policy approaches to raise funds lessons may be learnt from other countries such as Australia, Thailand, Vietnam and Korea who have implemented this system for health promotion [10-12]. Australia have been leaders in establishing and administering a tobacco control funding. The Victorian Health Promotion Foundation (VicHealth), established in 1987, was the first foundation to be funded by a tax on tobacco with a legislative mandate to promote health in the state of Victoria, Australia [20, 21]. It was regarded an inspiration [20, 22] and subsequently, led to the establishment of the West Australian Health Promotion Foundation (Healthway) [20]. Relevant here is that VicHealth is a self-governing statutory board enabling it to be an independent board. This independence allows the Foundation to distance themselves from tobacco industry influence.

Similar to VicHealth, the Thai Health Promotion foundation (ThaiHealth) is a self-governing statutory board, funded by industry money but independent from tobacco industry interference [20].

For peer review only - http://bmjopen.bmj.com/site/about/guidelines.xhtml

Revenue for ThaiHealth was established from a new 2% earmarked tax on tobacco and alcohol importers and manufacturers to support tobacco control and health promotion efforts [23]. Vietnam and Korea have also adopted similar funding models [24] mobilising financial resources to strengthening cessation services and develop interventions to help tobacco growers change their occupations [20, 25, 26].

In the UK context, there was good agreement was that the fund should be focused on tackling smoking-related health inequalities and that preventing people from starting to smoke and helping them to quit rather than treating smoking-related disease. Experts in this current study also suggested that the fund should be ring-fenced and allocated yearly with a distribution at local, regional, and national levels to support a comprehensive tobacco control plan towards meeting government targets. This is similar to the way VicHealth operate where their goals are aligned with government targets for example the 10-year goal that 400,000 more Victorians would be tobacco-free by 2023 [27]. Experts in this study also identified that the fund should be run in an independent and transparent way without any interference or input from the tobacco industry as VicHealth and ThaiHealth have done.

Several limitations in this study are worth noting. The qualitative nature of data offers depth of opinion within the research sample but does not offer any predictions about the frequency of specific stances within any wider population. As such, the value of qualitative policy research is in identifying useful reasoning and novel ideas, not making generalisations about how commonplace specific opinions are. This study was also affected by certain limitations inherent to policy research. The complexity of policies and policymaking environments is such that transferring learning from one policy to a different policy to a different policy is challenging [28]. For example, the US tobacco MSA may contain valuable lessons for tobacco control policy in the UK, but the importance of the differences in time periods and legislative contexts cannot be discounted. As such, few participants possessed the breadth of context and knowledge to be able to present comprehensive recommendations for policy. More commonly, participants presented in depth knowledge in specific areas or general principles for policymaking. However, this study offers new insights into an under-researched area. While the interviews were valuable in producing rich individual accounts into

For peer review only - http://bmjopen.bmj.com/site/about/guidelines.xhtml

relevant aspects of tobacco control, the key benefit of the discussion groups were in creating informed dialogue between experts. Together this data offered a valuable a means of arriving at grounded policy recommendations through interdisciplinary discussion, useful in policy research due to the extent to which policy is constructed through the discursive engagement of different coalitions of policy actors [29]. Another strength was using online data collection which proved to be straightforward reduced geographical barriers to participation among world leading experts in the UK, the US, and South Africa.

Conclusion

Smoking remains a leading preventable cause of death and disease in the UK with much of this impacting the poorest communities. The UK is a leader in tobacco control and the implementation of a tobacco control fund would further enrich this status and help meet the proposed government tobacco free targets. However, there is no 'one size fits all' template for such fund, the structure and operations of the fund would need to adapt to other countries to fit the culture, government ideology, and social context. This research shows that experts support the introduction of a tobacco control fund to reduce inequalities in health and achieve its target of a tobacco free generation by 2034. It provides early insights into how a fund might be established and administered in the UK and sets out key foundational principles that must be engaged with in designing a tobacco control fund policy in the UK. Importantly, although there was no one funding approach had unanimous support, experts agreed that establishing an 'imperfect policy' that provides dedicated funding is preferable to delay and inaction.

Contributions: CB and CP conducted all of interviews and led the analysis. SH wrote the first draft of the manuscript. All authors read and approved the final manuscript.

Competing interests: No conflicts of interest.

For peer review only - http://bmjopen.bmj.com/site/about/guidelines.xhtml

Funding: This work was support by the Medical Research Council (MC_UU_00022/2 and (MC_UU_12017/15), Chief Scientist Office of the Scottish Government Health Directorates (SPHSU15 and SPHSU17) and Cancer Research UK Grant PPRCTAGPJT\100003.

Data sharing statement: All data relevant to the study are included in the article or uploaded as supplementary information.

Ethics Approval statement: Ethical approval for this study was granted by the College of Social Sciences Research Ethics Committee at the University of Glasgow (reference number: 400190213).

For peer review only

References

1. WHO. Tobacco: Leading cause of death, illness and impoverishment 2021 [Available from: https://www.who.int/en/news-room/fact-sheets/detail/tobacco.
2. ASH. Written evidence submitted by Action on Smoking and Health. 2020.
3. Branston JR and Gilmore A. The extreme profitability of the UK tobacco market and the rationale for a new tobacco levy. University of Bath; 2015.
4. Branston JR and Gilmore AB. The failure of the UK to tax adequately tobacco company profits. 2019;42(1):69-76.10.1093/pubmed/fdz004
5. Cancer Research UK. Tobacco statistics 2020 [Available from: https://www.cancerresearchuk.org/health-professional/cancer-statistics/risk/tobacco.
6. Office for National Statistics. Adult smoking habits in the UK: 2019. 2020.
7. HM Government. Advancing our health: prevention in the 2020s – consultation document 2019 [Available from: https://www.gov.uk/government/consultations/advancing-our-health-prevention-in-the-2020s/advancing-our-health-prevention-in-the-2020s-consultation-document.
8. The Scottish Government. Creating a Tobacco-Free Generation: A Tobacco Control Strategy for Scotland. Edinburgh: The Scottish Government; 2013.
9. Cancer Intelligence Team. Smoking prevalence projections for England, Scotland, Wales and Northern Ireland, based on data to 2018/19: Cancer Research UK; 2020 [
10. Nam EW, Hasegawa T, Davies JK, et al. Health promotion policies in the Republic of Korea and Japan: a comparative study. 2006;13(1):20-8
11. Smith KE, Savell E and Gilmore AB. What is known about tobacco industry efforts to influence tobacco tax? A systematic review of empirical studies. *Tob Control* 2013;22(2):144-53.https://dx.doi.org/10.1136/tobaccocontrol-2011-050098
12. Nguyen DT, Luong KN, Phan HT, et al. Cost-Effectiveness of Population-Based Tobacco Control Interventions on the Health Burden of Cardiovascular Diseases in Vietnam. 2021;33(8):854-60.10.1177/1010539521999873
13. HM Treasury. Tobacco levy: response to the consultation. HM Treasury; 2015.
14. HM Treasury. Tobacco levy: consultation. HM Treasury; 2014.
15. Scottish Government. Minimum Unit Pricing 2017 [Available from: http://www.gov.scot/Topics/Health/Services/Alcohol/minimum-pricing.
16. HM Revenue & Customs. Policy paper: Soft Drinks Industry Levy 2016 [Available from: https://www.gov.uk/government/publications/soft-drinks-industry-levy/soft-drinks-industry-levy.

For peer review only - http://bmjopen.bmj.com/site/about/guidelines.xhtml

17. Cancer Research UK. Cancer Research UK urges government to make big tobacco cough up 2016 [Available from: https://www.cancerresearchuk.org/about-us/cancer-news/press-release/2016-01-26-cancer-research-uk-urges-government-to-make-big-tobacco-cough-up.
18. Action on Smoking and Health. Big Tobacco should pay for the damage it does 2018 [Available from: https://ash.org.uk/media-and-news/blog/big-tobacco-should-pay-for-the-damage-it-does/.
19. Public Health England. Fiscal and pricing policies: evidence report and framework. 2018.
20. International Union Against Tuberculosis and Lung Disease. Sustainable Funding Models for Tobacco Control: a Discussion Paper. 2014.
21. Bonito S. VicHealth: The World's First Health Promotion Foundation. Michigan: University of Michigan; 2014.
22. Rechter J. Innovate, Inform, Integrate: VicHealth is transforming to meet innovation and the new health promotion challenges. 2015;30(3):413-7.10.1093/heapro/dav086
23. Hamann SL, Mock J, Hense S, et al. Building tobacco control research in Thailand: meeting the need for innovative change in Asia. *Health Res Policy Sys* 2012;10(1):3.10.1186/1478-4505-10-3
24. Decision on the establishment and approval of the regulation on the organization and operation of the Vietnam Tobacco Control Fund, 47/2013/QĐ-TTg (2013).
25. Law on Prevention and Control of Tobacco Harms, 09/2012/QH13 (2012).
26. National Tobacco Control Center. Tobacco taxation in Korea 2016 [Available from: https://nosmk.khealth.or.kr/ntcc/eng/subIndex/569.do.
27. VicHealth. VicHealth Tobacco Strategy 2019–2023 2019 [updated 25 February 2020. Available from: https://www.vichealth.vic.gov.au/media-and-resources/publications/vichealth-tobacco-strategy.
28. Cairney P. Complexity theory in political science and public policy. 2012;10(3):346-58
29. Herzog C and Ali C. Elite interviewing in media and communications policy research. 2015;11(1):37-54

For peer review only

BMJ Open

Experts' views on how to design a Tobacco Control Fund in the UK

Journal:	*BMJ Open*
Manuscript ID	bmjopen-2022-066224.R1
Article Type:	Original research
Date Submitted by the Author:	14-Nov-2022
Complete List of Authors:	Hilton , Shona; University of Glasgow MRC/CSO Social and Public Health Sciences Unit<br>Smith, Marissa; University of Glasgow MRC/CSO Social and Public Health Sciences Unit<br>Buckton, Christina; University of Glasgow MRC/CSO Social and Public Health Sciences Unit<br>Patterson, Chris; University of Glasgow MRC/CSO Social and Public Health Sciences Unit
**Primary Subject Heading**:	Public health
Secondary Subject Heading:	Health policy, Health services research, Smoking and tobacco
Keywords:	PUBLIC HEALTH, QUALITATIVE RESEARCH, Health policy < HEALTH SERVICES ADMINISTRATION & MANAGEMENT

SCHOLARONE™ Manuscripts

I, the Submitting Author has the right to grant and does grant on behalf of all authors of the Work (as defined in the below author licence), an exclusive licence and/or a non-exclusive licence for contributions from authors who are: i) UK Crown employees; ii) where BMJ has agreed a CC-BY licence shall apply, and/or iii) in accordance with the terms applicable for US Federal Government officers or employees acting as part of their official duties; on a worldwide, perpetual, irrevocable, royalty-free basis to BMJ Publishing Group Ltd ("BMJ") its licensees and where the relevant Journal is co-owned by BMJ to the co-owners of the Journal, to publish the Work in this journal and any other BMJ products and to exploit all rights, as set out in our licence.

The Submitting Author accepts and understands that any supply made under these terms is made by BMJ to the Submitting Author unless you are acting as an employee on behalf of your employer or a postgraduate student of an affiliated institution which is paying any applicable article publishing charge ("APC") for Open Access articles. Where the Submitting Author wishes to make the Work available on an Open Access basis (and intends to pay the relevant APC), the terms of reuse of such Open Access shall be governed by a Creative Commons licence – details of these licences and which Creative Commons licence will apply to this Work are set out in our licence referred to above.

Other than as permitted in any relevant BMJ Author's Self Archiving Policies, I confirm this Work has not been accepted for publication elsewhere, is not being considered for publication elsewhere and does not duplicate material already published. I confirm all authors consent to publication of this Work and authorise the granting of this licence.

For peer review only

Experts' views on how to design a Tobacco Control Fund in the UK

Shona Hilton[1], Marissa J. Smith[1*], Christina Buckton[1], and Chris Patterson[1]

[1] MRC/CSO Social and Public Health Sciences Unit, University of Glasgow, Glasgow, UK

*Corresponding author

Email: Marissa.Smith@glasgow.ac.uk Tel: (+44) 0141 353 7500

Keywords: Public policy, Tobacco industry, Health services

Word count: 3500

For peer review only

Abstract

Objective

To explore expert views on the potential value, and approaches to establishing and administering a tobacco control fund in the United Kingdom (UK).

Design

Semi-structured interviews and follow-up discussion groups.

Subjects

Twenty-four UK and international experts on tobacco control regulation, public health, economics, or law from the academic, public, private and third sector.

Methods

Participants considered the relative merit of 1) general excise tax on retail tobacco sales; 2) ring-fenced hypothecation of excise taxes on retail tobacco sales; and 3) a direct levy on tobacco manufacturers. Preliminary synthesis of interview findings was deliberated upon in two follow-up discussion groups to identify key considerations for policy design.

Result

Most experts agreed that a ring-fenced tobacco control fund would be a valuable method of raising predictable and reliable funds from tobacco producers either using either companies' sales volume or market share as a way to establish the proportion they should pay. Experts predominantly recommended that a fund in the UK should be administered by a government body with devolved nation input and with an independent advisory group. They typically indicated that funding should be allocated yearly with distribution at local, regional, and national levels to support smoking prevention and cessation rather than treatment activities with priority given to measures that tackle smoking-related inequalities.

Conclusion

For peer review only - http://bmjopen.bmj.com/site/about/guidelines.xhtml

There was overwhelming agreement by experts on the need to establish a tobacco control fund to help meet the proposed government tobacco-free targets to reduce adult smoking prevalence to 5% by 2030 (UK) and 2034 (Scotland).

Strengths and limitations of this study

- Methodology includes semi-structured interviews with 24 UK and international experts on tobacco control regulation, public health, economics, or law from the academic, public, private and third sector, facilitating understandings of the potential value of a tobacco control fund in the UK.
- Follow-up discussion groups created informed dialogue between experts to collaboratively identify key considerations for policy design in this area by bringing together groups of policy actors diverse in terms of their specific areas of expertise and the sectors within which they have professional experience.
- Quantitative thematic analysis of the data allows depth of opinions but cannot offer predictions about the frequency of specific opinions with a wider population.
- The policy research offered new insights into an under-research area, but the complexity of policies and policymaking environments is such that transferring learning from one policy to a different policy is challenging.

For peer review only - http://bmjopen.bmj.com/site/about/guidelines.xhtml

Introduction

Worldwide tobacco kills more than 8 million people per annum [1] including nearly 100,000 preventable deaths in the UK [2]. Tobacco is highly addictive, there is no safe level of exposure and all forms of tobacco are harmful to health increasing the risk of cancers, heart disease, and other NCDs [1]. Despite a broad range of effective tobacco control policies, the tobacco trade continues to be highly profitable [3]. In contrast, the economic costs of tobacco use in society are greater than the costs for treating tobacco-related diseases. For example in the UK, revenue from excise duty on tobacco sales continues to be substantially lower than the health costs of smoking [4]. While UK smoking prevalence has declined precipitously in response to tobacco control action [5, 6], the smoking inequality gap has grown [6] as smoking contributes to poverty by diverting household spending from basic needs such as food and shelter to tobacco.

To further reduce smoking, the Westminster and Scottish governments have proposed targets to reduce adult smoking prevalence to 5% by 2030 and 2034, respectively [7, 8]. However, for these proposed targets to be met prevalence rates need to decline at a much faster rate [9] which may require additional tobacco control measures.

One policy option that has been proposed is to establish a ring-fenced tobacco control fund. This system for health promotion has been pursued in other countries including Australia, Vietnam, Korea, and Thailand [10, 11]. In the UK in 2015, Her Majesty's (HM) Treasury published their conclusions on an earlier consultation on the potential design of a levy on tobacco manufacturers and importers [12]. The consultation considered a tobacco levy under the administration of the existing corporation tax system, imposed on manufacturers and importers of products on which tobacco excise duty is paid [13]. Whilst the proposal received the support of a broad range of health charities, professional bodies, and academics; the UK Government decided not to pursue the tobacco levy, citing concerns that costs would be passed on to consumers and that tobacco sales are already subject to escalating duties [13]. Since the government rejected this tobacco industry levy, other fiscal approaches to tackling the harms caused by unhealthy products have been introduced, including Scotland's Minimum Unit Pricing for alcohol [14], and the 'soft hypothecation' of the UK's Soft Drinks Industry

For peer review only - http://bmjopen.bmj.com/site/about/guidelines.xhtml

Levy (SDIL) [15]. In light of an increasing political willingness to implement other fiscal interventions, and the continued advocacy for a tobacco control fund from the public health community [16, 17], raising revenue for a tobacco control fund from the tobacco industry remains a viable policy option. This paper explores contemporary views of UK and international tobacco control and public health experts on the potential value of, and approaches to establishing and administering a tobacco control fund. In doing so we identify key considerations for its design.

Methods

Interviews

We developed a purposive sampling frame to target UK and international experts in tobacco control regulation, public health, economics, or law from the academic, public, private and third sector. Twenty-four experts agreed to participate after reading the participant information sheet, privacy notice and signing the consent form. Eighteen were based in the UK, four in the United States, and two in South Africa. Table 1 illustrates the distribution of participants by the sector in which they primarily worked and their principal topic of work. Participants classified as ‘public health’ were those who work and publish in the broader area of public health. Participants classified as ‘tobacco control’ were those who had expertise in the area of tobacco control and predominately work and publish in the tobacco control area.

A semi-structured interview schedule (Appendix A) was informed by reviewing international academic and grey literature on tobacco control funds. The interviews were conducted between September 2020 and January 2021 by CP and CB. One interview was conducted by telephone and the remaining 23 interviews were conducted using Microsoft Teams video meetings. The interviews lasted between approximately 45 and 60 minutes, all were recorded and transcribed verbatim.

For peer review only

121 **Table 1: Sample composition by primary sector of work and primary area of expertise**

**Primary sector**	**Professional disciplinary approach to tobacco control**			**Total**
	Economics / Law	Public Health	Other	
Academia	6	3	0	**9**
Public sector	0	4	3	**7**
Third sector	1	1	5	**7**
Private sector	1	0	0	**1**
**Total**	**8**	**8**	**8**	**24**

122

123 **Discussion Groups**

124 In March 2021, two follow-up online discussion groups were conducted by CB and CP with nine
125 individuals using Microsoft Teams. Participants were selected for these follow-ups based on their
126 sectorial expertise and to represent key disciplines. The first discussion group (n=5) included three
127 third sector professionals with expertise spanning tobacco control and public health advocacy and two
128 academic economists. The second discussion group (n=4) included two public sector professionals
129 with roles in tobacco control and public health policy and two academics with expertise in law and
130 public health. The aim of these groups was to consider the synthesis of views from the interviews on
131 the potential value of a tobacco control fund and to identify key considerations for policy design. Each
132 discussion group lasted two hours, and group discussions were recorded for later checking against the
133 minutes.

134

135 **Analysis**

For peer review only

We conducted thematic analysis of the data form the interview transcripts and discussion group minutes. The process followed Braun and Clarke's [18] six-phase framework for thematic analysis. The research team read and re-read the transcripts to become familiar with the data, and then iteratively constructed a coding frame to enable consistent organisation of relevant data. NVivo was used to organise categories on the basis of inductive themes that emerged from close reading of the, capture of both areas of agreement and less typical perspectives across a range of categories. The discussion group recordings and minutes were cross-compared with the interview coding frame to confirm and expand on codes relating to recommendations for policy design of a tobacco control fund. To maintain participant anonymity as agreed at consent, brackets have been used to replace identifiable details about professional activities.

Where appropriate, the number of participants that gave specific opinions are presented as counts and proportions to help illustrate the balance of opinion with the sample. However, it must be noted that, given the qualitative methodology used in this study, these numbers cannot necessarily be generalised to any wider population.

Ethical approval for this study was granted by the College of Social Sciences Research Ethics Committee at the University of Glasgow (reference 400190213).

Patient and Public Involvement

None.

Results

The results are presented in accordance with the inductive coding categories developed during the analysis stage.

For peer review only

For peer review only - http://bmjopen.bmj.com/site/about/guidelines.xhtml

What is the potential value of a tobacco control fund?

There was general agreement that a tobacco control fund could be a valuable revenue for raising predictable and reliable funds direct from the tobacco industry. Typically, this was viewed as a way to boost current public health efforts:

> *"The more money that we can earmark, ring-fence into public health and tobacco control efforts the better from a public health point of view." (P02, academic, law)*

However, two participants cautioned that whilst such a fund was largely welcomed, it would be important that a tobacco control fund did not act as a disincentive for government funding or cutbacks to existing tobacco control activities. Participants also welcomed the fact that an industry-funded payment, would help hold the tobacco industry more accountable for the damage they cause to society, with one participant stating: "*There's some sort of nice symmetry about money from the tobacco industry being used to improve or solve some of the problems it creates" (P05, third sector, public health)*. However, participants also warned that the funding mechanism of extracting money from the tobacco industry would need careful consideration so that the levy was not passed on to nicotine-dependent and socially deprived smokers, with one participant warning that:

> *"It doesn't really make sense, I think, to pursue further interventions that actually further widen the health inequality that we have." (P07, academic, public health)*

How might a tobacco control fund be designed?

Participants considered in more detail how a tobacco control fund might be designed to raise funds: 1) general excise tax on retail tobacco sales; 2) ring-fenced hypothecation of excise taxes on retail tobacco sales; and 3) a direct levy on tobacco manufacturers. Participants were asked to consider the relative merits of each funding approach.

For peer review only - http://bmjopen.bmj.com/site/about/guidelines.xhtml

General excise tax on retail tobacco sales

Participants were widely supportive of excise taxes, predominantly for their role in decreasing consumption, but also for their role in fundraising with some participants drawing on excise tax in Australia and New Zealand as useful models for the UK policymakers to consider. Participants highlighted the simplicity, efficiency, and political acceptability of excise tax as positive attributes of this approach. Some participants expressed doubt about the usefulness of excise taxes to fundraising given falling revenue with one academic stating:

> *"The UK I know is now sort of sitting at the top of that revenue situation where they increased excise tax, revenues are not increasing all that much because the excise taxes are very high already." (P01, academic, economics)*

In contrast, other participants suggested that the government can effectively control the extent to which taxes are passed on to consumers by capping retail prices, meaning that increasing specific excise tax can raise revenue while ensuring that retail prices do not increase so consumers do not bear the additional cost [19].

Ring-fenced hypothecated excise tax

Participants discussed the potential for some or all of excise taxation on retail tobacco sales to be hypothecated, meaning that it would be diverted into a specific fund instead of general government funds. This approach was viewed by participants as publicly acceptable as explained:

> *"Dealing with the consequences or addressing the harms that arise from the product I think is actually instinctively appealing to people." (P22, public sector, public health)*

However, while appealing in principle, participants overwhelmingly indicated that hypothecation would meet with too much opposition from HM Treasury, as noted by one academic:

> *"Politicians in general don't like it, they're very particular about being elected to*

For peer review only - http://bmjopen.bmj.com/site/about/guidelines.xhtml

do the right thing, and they wish to retain their independence and their freedom for manoeuvre. So, it can be a challenging negotiation that one." (P03, academic, public health)

Despite this view, participants identified the UK's SDIL as a possible route to hypothecation but noted that the funds raised by SDIL were not ultimately ring-fenced for the purposes they originally presented to the public, with one participant noting:

"The sugar tax was pushed through with major public support on the basis of hypothecation. And then guess what? There was a crisis and the money, the revenues raised for the sugar tax miraculously didn't get spent on children's breakfast clubs and school sports but have been used to fill gaps in broader public health, and possibly NHS budgets as well. That's always a risk". (P03, academic, public health)

This led to discussions about how to win political support for hypothecation and the merits of creating a general health fund instead of a tobacco control fund. As explained by one participant:

"I absolutely expect that it would be easier to convince policymakers, who generally don't like hypothecated taxes, [of the merits of a general health fund] so the more freedom that you give them, the more acceptable it's likely to be. But I would rather think it probably would be less acceptable to the public, because … if you're using the sort of, polluter pays type principle, then, you know, people expect that there is a direct consequence between those two things." (P22, public sector, public health)

Another potential route to hypothecation discussed that bypassed HM Treasury was:

"If it was seen as a user fee done by the Department of Health and Social Care, then it would bypass the treasury's normal functioning." (P09, academic,

For peer review only - http://bmjopen.bmj.com/site/about/guidelines.xhtml

economics)

Bypassing HM Treasury was advocated by three participants who suggested taking inspiration from the UK's Pharmaceutical Price Regulation Scheme (PPRS) [20, 21], through which the Department for Health and Social Care (DHSC) (and not the treasury) receives excess profits from participating pharmaceutical companies and uses that funding to address shortfalls in NHS budgets due to expenditure on novel treatments. While this scheme is not an example of hypothecated tax, it was presented as a precedent for the DHSC receiving funds from industry, and an illustration of relevant administrative expertise within the DHSC, as explained:

> *"The important thing from the tobacco point of view is, you've established this principle of soft hypothecation where the rebates from the industry go back specifically for…or back to the [DHSC], rather just the Treasury who just grab it." (P24, private sector, pharmaceuticals)*

A levy on the tobacco industry

Participants who favoured this approach (n=22, 92%) typically viewed it as a means to extract funds from industry instead of from consumers, which may be more appealing to the public and could help convince policymakers:

> *"I think politically it's more sellable to the public [than excise tax increases]." (P08, third sector, tobacco control)*

> *"I think that would be a decision-making factor for any governmental policy measure that got put forward, that it would be very much clear that the industry would be the contributor, not the public, if you like." (P22, public sector, public health)*

Controlling retail prices was deemed an essential part of ensuring that the cost of a levy is borne by the industry.

For peer review only - http://bmjopen.bmj.com/site/about/guidelines.xhtml

257 *"The way that the tobacco companies are monopolies and making excess profits is*
258 *because they are using gradual escalator duty increases to increase their own*
259 *prices. So, you need to cap prices" (P24, private sector, pharmaceuticals).*

260 Conversely, some participants argued that retail prices should not be limited as price increases are
261 beneficial in reducing consumption: As explained:

262 *"When you do see tax increases, you tend to see over-shifting of the tax and using*
263 *that as an opportunity to raise price and capitalise on at least the addicted*
264 *consumers that are still in the market. So that is happening, but I don't know that*
265 *that's necessarily a bad thing, because in the end those price increases are also*
266 *very effective and leading to additional cessation and particularly in terms of*
267 *preventing initiation." (P04, academic, economics)*

268 The PPRS was presented as a potential model for extracting industry profits outside of excise taxes,
269 and one that has been refined over many years to limit potential loopholes. However, PPRS was
270 generally considered to be of limited use in having real-world transferability from pharmaceuticals to
271 tobacco. One public sector participant stated:

272 *"The UK pharmaceutical market's status as a virtual monopsony differs starkly*
273 *from the tobacco industry and suggested that such a scheme may incentivise the*
274 *lowering of tobacco prices." (P14, public sector, tobacco control)*

275 In thinking about general principles of where the tobacco control fund might come from, participants
276 discussed considered three options from 'profits', 'sales volume', or 'market share'. The option of a
277 payment coming directly from 'profits' was largely discounted on the grounds that:

278 *"Multinational companies are very good at moving money around and shifting*
279 *profits to other countries with lower tax systems." (P03, academic, public health)*

For peer review only - http://bmjopen.bmj.com/site/about/guidelines.xhtml

The option of using ‘sales volume’ or ‘market share’ were both more popular as they were deemed more difficult for companies to obscure and shift money. Examples given were: “*The harm is linked to the sales volume of the product, not to the profits they make from it” (P23, third sector, tobacco control).* Or that: “*Market share is the easiest way to do it. And you may want to average market share over the past 30 years or something like that to try to figure out what it is” (P02, academic, law).*

Other policy design considerations

After considering the different options for designing a tobacco control fund, participants considered other factors that would be essential for gaining public and political support. A key factor identified was the need for the fund to be administered by a government body with an independent advisory group to ensure transparent decision-making. As highlighted by one participant a requirement would be:

> *“A transparent body that both industry and [academic] researchers and the government had trust in to operate transparently and fairly and not be unduly influenced by any stakeholders, you just need to make it an independent body.” (P05, third sector, economist)*

It was also agreed that the fund should be allocated yearly with distribution at local, regional, and national levels to support smoking prevention and cessation rather than treatment activities with priority given to tackling smoking-related inequalities in the most deprived areas. This was deemed important for:

> *“Making smoking obsolete, to massively benefit the most deprived communities both economically as well as in health terms.” (P18, public sector, public health)*

For peer review only

Discussion

Experts considered three broad approaches to raising funds: raising existing excise tax on tobacco sales, introducing a hypothecated excise tax, and a tobacco industry levy. Each approach was assessed as having strengths and weaknesses, for example, raising excise taxes was seen as politically feasible and administratively simple, while hypothecation was seen as least politically plausible due to potential Treasury resistance and a tobacco levy was deemed as a logical advocacy route following the polluter pays principle to ensure the industry pays for its damage to society. Experts agreed that whichever mechanism is chosen must be clearly guided by what the fund is directly trying to achieve. This is consistent with a recent Public Health England report on fiscal and pricing policies [22], which highlights that policy success depends on the clarity of policy goals. Most experts agreed that key principles underlying the design of a fund would be to collect predictable and reliable funds from transnational tobacco producers either from companies' sales volume or their market share as a way to assign responsibility and establish the proportion they should pay. There was agreement that any fundraising mechanism which extracts funds from industry and avoids the potentially regressive effects of price increases on consumers may be the optimal fundraising approach. However, there was acknowledgement that policy goals have trade-offs. For example to achieve both health promotion and revenue-raising objectives is possible within the same policy when demand for a product is relatively price-inelastic, as is the case with tobacco [22]. From this perspective, permitting costs to be passed on to customers and ensuring that costs are paid by industry may each be valid goals, and designing the policy requires skill. The implementation of other fiscal interventions to tackle the harms caused by unhealthy products, such as the SDIL, our research has shown the political willingness to establish a tobacco control fund. Experts described the potential for resistance from the Treasury, politicians and the public to these three potential tobacco control fund proposals. Industry resistance and influence is relevant in terms of both policy acceptability and ensuring compliance with World Health Organisation (WHO) Framework Convention on Tobacco Control (FCTC) [23], thus future research could explore the potential for resistance from industry actors concerning a the design of a tobacco control fund in the UK.

For peer review only

In considering the policy approaches to raise funds lessons may be learned from other countries such as Australia, Thailand, Vietnam, and Korea who have implemented this system for health promotion [10, 11, 24]. Australia have been leaders in establishing and administering tobacco control funding. The Victorian Health Promotion Foundation (VicHealth), established in 1987, was the first foundation to be funded by a tax on tobacco with a legislative mandate to promote health in the state of Victoria, Australia [25, 26]. The levy was set at 5% and this increased the state tobacco licence fee from 25% to 30%. In the first year, the money raised approximately $23 million and this was paid directly into the foundation [27]. It was regarded an inspiration [25, 28] and subsequently, led to the establishment of the West Australian Health Promotion Foundation (Healthway) [25]. Relevant here is that VicHealth is a self-governing statutory board enabling it to be an independent board. This independence allows the Foundation to distance themselves from tobacco industry influence.

Similar to VicHealth, the Thai Health Promotion Foundation (ThaiHealth) is a self-governing statutory board, funded by industry money but independent from tobacco industry interference [25]. Revenue for ThaiHealth was established from a new 2% earmarked tax on tobacco and alcohol importers and manufacturers to support tobacco control and health promotion efforts [29]. Vietnam and Korea have also adopted similar funding models [30] mobilising financial resources to strengthen cessation services and develop interventions to help tobacco growers change their occupations [25, 31, 32].

In the UK context, there was good agreement that the fund should be focused on tackling smoking-related health inequalities and preventing people from starting to smoke and helping them to quit rather than treating smoking-related diseases. Experts in this current study also suggested that the fund should be ring-fenced and allocated yearly with distribution at local, regional, and national levels to support a comprehensive tobacco control plan towards meeting government targets. This is similar to the way VicHealth operate where their goals are aligned with government targets for example the 10-year goal that 400,000 more Victorians would be tobacco-free by 2023 [33]. Experts in this study also identified that the fund should be run in an independent and transparent way without any interference or input from the tobacco industry as VicHealth and ThaiHealth have done.

For peer review only - http://bmjopen.bmj.com/site/about/guidelines.xhtml

Several limitations in this study are worth noting. The qualitative nature of data offers depth of opinion within the research sample but does not offer any predictions about the frequency of specific stances within any wider population. We were satisfied that the diversity of professional experience and expertise across these 24 participants provided us with a sample that represented the breadth of perspectives likely to be found within our target population. The value of qualitative policy research is in identifying useful reasoning and novel ideas, not making generalisations about how commonplace specific opinions are. This study was also affected by certain limitations inherent to policy research. The complexity of policies and policymaking environments is such that transferring learning from one policy to a different policy is challenging [34]. For example, the US tobacco Master Settlement Agreement (MSA) may contain valuable lessons for tobacco control policy in the UK, but the importance of the differences in time periods and legislative contexts cannot be discounted. As such, few participants possessed the breadth of context and knowledge to be able to present comprehensive recommendations for policy. More commonly, participants presented in-depth knowledge in specific areas or general principles for policymaking. However, this study offers new insights into an under-researched area. While the interviews were valuable in producing rich individual accounts into relevant aspects of tobacco control, the key benefit of the discussion groups was in creating an informed dialogue between experts. Together this data offered a valuable means of arriving at grounded policy recommendations through interdisciplinary discussion, useful in policy research due to the extent to which policy is constructed through the discursive engagement of different coalitions of policy actors [35]. Another strength was using online data collection which proved to be straightforward reduced geographical barriers to participation among world-leading experts in the UK, the US, and South Africa.

Conclusion

Smoking remains a leading preventable cause of death and disease in the UK with much of this impacting the poorest communities. The implementation of a tobacco control fund would help meet the proposed government tobacco-free targets. However, there is no ‘one size fits all’ template for

For peer review only - http://bmjopen.bmj.com/site/about/guidelines.xhtml

such fund, the structure, and operations of the fund would need to adapt to other countries to fit the culture, government ideology, and social context. This research shows that experts support the introduction of a tobacco control fund to reduce inequalities in health and achieve the English and Scottish targets of reducing adult smoking prevalence to 5% by 2030 and 2034, respectively. It provides early insights into how a fund might be established and administered in the UK and sets out key foundational principles that must be engaged with in designing a tobacco control fund policy in the UK. Importantly, although there was no one funding approach had unanimous support, experts agreed that establishing an 'imperfect policy' that provides dedicated funding is preferable to delay and inaction.

Contributions: **Shona Hilton:** Conceptualisation, Data Curation, Methodology, Validation, Writing-Original draft preparation. **Marissa J. Smith:** Data Curation, Visualisation, Writing - Review & Editing. **Christina Buckton:** Conceptualisation, Data Curation, Methodology, Investigation, Validation, Writing - Review & Editing. **Chris Patterson:** Conceptualisation, Data Curation, Methodology, Investigation, Validation, Writing - Review & Editing.

Competing interests: No conflicts of interest.

Funding: This work was supported by the Medical Research Council (MC_UU_00022/2 and (MC_UU_12017/15), Chief Scientist Office of the Scottish Government Health Directorates (SPHSU15 and SPHSU17) and Cancer Research UK Grant PPRCTAGPJT\100003.

Data sharing statement: All data relevant to the study are included in the article or uploaded as supplementary information.

Ethics Approval statement: Ethical approval for this study was granted by the College of Social Sciences Research Ethics Committee at the University of Glasgow (reference number: 400190213).

For peer review only - http://bmjopen.bmj.com/site/about/guidelines.xhtml

References

1. WHO. Tobacco: Leading cause of death, illness and impoverishment 2021 [Available from: https://www.who.int/en/news-room/fact-sheets/detail/tobacco.
2. ASH. Written evidence submitted by Action on Smoking and Health. 2020.
3. Branston JR, Gilmore A. The extreme profitability of the UK tobacco market and the rationale for a new tobacco levy. University of Bath; 2015.
4. Branston JR, Gilmore AB. The failure of the UK to tax adequately tobacco company profits. 2019;42(1):69-76.
5. Cancer Research UK. Tobacco statistics 2020 [Available from: https://www.cancerresearchuk.org/health-professional/cancer-statistics/risk/tobacco.
6. Office for National Statistics. Adult smoking habits in the UK: 2019. 2020.
7. HM Government. Advancing our health: prevention in the 2020s – consultation document 2019 [Available from: https://www.gov.uk/government/consultations/advancing-our-health-prevention-in-the-2020s/advancing-our-health-prevention-in-the-2020s-consultation-document.
8. The Scottish Government. Creating a Tobacco-Free Generation: A Tobacco Control Strategy for Scotland. Edinburgh: The Scottish Government; 2013.
9. Cancer Intelligence Team. Smoking prevalence projections for England, Scotland, Wales and Northern Ireland, based on data to 2018/19: Cancer Research UK; 2020 [
10. Nam EW, Hasegawa T, Davies JK, Ikeda N. Health promotion policies in the Republic of Korea and Japan: a comparative study. 2006;13(1):20-8.
11. Nguyen DT, Luong KN, Phan HT, Tran AT, Dao ST, Poudel AN, et al. Cost-Effectiveness of Population-Based Tobacco Control Interventions on the Health Burden of Cardiovascular Diseases in Vietnam. APJPH. 2021;33(8):854-60.
12. HM Treasury. Tobacco levy: response to the consultation. HM Treasury; 2015.
13. HM Treasury. Tobacco levy: consultation. HM Treasury; 2014.
14. Scottish Government. Minimum Unit Pricing 2017 [Available from: http://www.gov.scot/Topics/Health/Services/Alcohol/minimum-pricing.
15. HM Revenue & Customs. Policy paper: Soft Drinks Industry Levy 2016 [Available from: https://www.gov.uk/government/publications/soft-drinks-industry-levy/soft-drinks-industry-levy.
16. Cancer Research UK. Cancer Research UK urges government to make big tobacco cough up 2016 [Available from: https://www.cancerresearchuk.org/about-us/cancer-news/press-release/2016-01-26-cancer-research-uk-urges-government-to-make-big-tobacco-cough-up.
17. Action on Smoking and Health. Big Tobacco should pay for the damage it does 2018 [Available from: https://ash.org.uk/media-and-news/blog/big-tobacco-should-pay-for-the-damage-it-does/.
18. Braun V, Clarke V. Thematic analysis. APA handbook of research methods in psychology, Vol 2: Research designs: Quantitative, qualitative, neuropsychological, and biological. APA handbooks in psychology®. Washington, D.C., USA: American Psychological Association; 2012. p. 57-71.
19. Branston JR, Gilmore AB. The case for Ofsmoke: the potential for price cap regulation of tobacco to raise £500 million per year in the UK. Tob Control. 2014;23(1):45-50.
20. Collier J. The pharmaceutical price regulation scheme. BMJ. 2007;334(7591):435-6.
21. Rodwin MA. How the United Kingdom Controls Pharmaceutical Prices and Spending: Learning From Its Experience. 2021;51(2):229-37.
22. Public Health England. Fiscal and pricing policies: evidence report and framework. 2018.
23. World Health Organisation. The WHO framework convention on tobacco control: an overview. Geneva, Switzerland: World Health Organisation; 2015.
24. Smith KE, Savell E, Gilmore AB. What is known about tobacco industry efforts to influence tobacco tax? A systematic review of empirical studies. Tob Control. 2013;22(2):144-53.
25. International Union Against Tuberculosis and Lung Disease. Sustainable Funding Models for Tobacco Control: a Discussion Paper. 2014.
26. Bonito S. VicHealth: The World's First Health Promotion Foundation. Michigan: University of Michigan; 2014.
27. Victorian Health Promotion Foundation. The Story of VicHealth. Carlton, Australia; 2005.

For peer review only - http://bmjopen.bmj.com/site/about/guidelines.xhtml

28. Rechter J. Innovate, Inform, Integrate: VicHealth is transforming to meet innovation and the new health promotion challenges. 2015;30(3):413-7.
29. Hamann SL, Mock J, Hense S, Charoenca N, Kungskulniti N. Building tobacco control research in Thailand: meeting the need for innovative change in Asia. Health Res Policy Sys. 2012;10(1):3.
30. Decision on the establishment and approval of the regulation on the organization and operation of the Vietnam Tobacco Control Fund, 47/2013/QĐ-TTg (2013).
31. Law on Prevention and Control of Tobacco Harms, 09/2012/QH13 (2012).
32. National Tobacco Control Center. Tobacco taxation in Korea 2016 [Available from: https://nosmk.khealth.or.kr/ntcc/eng/subIndex/569.do.
33. VicHealth. VicHealth Tobacco Strategy 2019–2023 2019 [updated 25 February 2020. Available from: https://www.vichealth.vic.gov.au/media-and-resources/publications/vichealth-tobacco-strategy.
34. Cairney P. Complexity theory in political science and public policy. 2012;10(3):346-58.
35. Herzog C, Ali C. Elite interviewing in media and communications policy research. 2015;11(1):37-54.

For peer review only

Appendix A: Interview topic guide

Part 1: Introduction/background

- First of all, thank you for taking part in this research.
- I'm [researcher name], a research assistant at the University of Glasgow with an interest in the communication of health issues and policies.
- Your will be aware from the participant information sheet that this is a project funded by Cancer Research UK to collect expert views on the extent to which a direct levy on the tobacco industry (a so called 'polluter pays' levy) might be an effective tobacco control measure. We are looking to compare the different forms of a levy and their potential impacts; consider how funds generated could be used for tobacco control activities; learn from international case studies; and if there is support for the levy, to provide recommendations for the next steps in advocating for it. Cancer Research UK (CRUK) will use the outcome of this project to inform their future tobacco control policy strategy.
- We are interviewing a mix of stakeholders with relevant expertise including legal experts, economists, financial or tax advisors, industry experts or representative bodies, charities and harm-reduction groups and academics or researchers.

Key points for consent:

- Can I confirm that you have received the participant information sheet and signed and returned your consent form.
- Just to reiterate, your taking part is voluntary, and you are free to withdraw at any time without giving a reason, up until the point where the data is published in an NHS Health Scotland report.
- Your participation will be anonymous. Excerpts from the interview may be quoted verbatim in a report and a paper, but quotations will be anonymised to avoid accidentally disclosing your identity. We realise that within certain policy communities it may be possible for others to identify you from your experiences of specific policies, as such we will take care to anonymise quotations as appropriate to avoid accidental disclosure. Non-anonymised interview recordings and transcripts will be destroyed securely upon completion of this research, but anonymised transcripts and consent forms will be stored securely by the University of Glasgow for a period of 10 years for the purposes of ensuring research integrity.
- Finally, the study has been approved by the Ethics Committee of the College of Social Sciences at the University of Glasgow.

Verbal confirmation of consent:

- Do you consent to take part in this research, and do you give consent for me to record this interview?
- Can you describe your professional role, and how it relates to taxation, tobacco control or the regulation of unhealthy commodities?

Part 2: How to raise funds? [Note: can focus on this more or less depending on interviewee's areas of expertise]

- What different mechanisms are you aware of for raising funds from the tobacco industry?
    - How about hypothecated excise taxes? [explain if necessary]

For peer review only - http://bmjopen.bmj.com/site/about/guidelines.xhtml

            - Do you think excise taxes are an effective away to raise revenue?
                - What are the pros and cons?
                - Could that revenue be effectively ring-fenced for specific purposes?
            - What specifically should a tax be applied to?
                - Sales at retail
                - Sales between manufacturers and distributors, or between distributors and retailers?
                - Sales crossing international boundaries
            - [If participant is favourable towards excise taxes]
                - Should tax rates differ between different tobacco products, such as heat not burn products, or should there be a flat rate of tax across all tobacco products?
                - Should taxes be direct (fixed amount per unit) or ad valorem (percentage of price)?
    - How about an industry levy? [explain if necessary – mandatory direct tax charged to the industry]
        - Are you aware of any direct taxes or levies that already exist?
            - Prompt: Soft drinks industry levy
            - Prompt: Pharmaceutical Price Regulation Scheme (UK)
            - Prompt: Tobacco product user fees (US)
    - Are there any examples of where other countries have used taxes or levies to raise funds from the tobacco industry for tobacco control? What lessons might we take from these?
    - Are there any novel approaches that should be considered?
        - Are there useful lessons to be learned from other pricing and taxation schemes (ECO, MUP, SDIL, single-use bag tax, Scottish Landfill Communities Fund)
- How far in advance should a fundraising scheme be planned?
    - Should funding rise over time, remain steady or drop over time?
    - At what point will it no longer be necessary?
    - What time of ongoing surveillance of the market will be necessary?
- What ways might the tobacco industry try to avoid contributing more? What are the implications of these strategies? How might we prevent these?
    - Prompt: TTCs 'hiding' profits in other countries
    - Prompt: over-shifting (increase prices on top of tax increases)
    - Prompt: under-shifting (absorb tax increases to keep retail prices stable
    - Prompt: brand-shifting (over- and under-shift different brands to encourage continued use and initiation of tobacco
    - Prompt: Collusion between TTCs to keep prices low
    - Prompt: Counter-marketing to undermine investment in public communication campaigns
    - Prompt: Subversion of a tobacco control fund idea eg: PMI's Tobacco Transition Fund
    - Prompt: Use of a TTF to gain access to policy making
    - Anything else?
- Is raising the price of tobacco products desirable? How does it relate to inequalities?
    - [If undesirable] Is capping prices a good idea? Is it practically and legally feasible?
    - Can you think of any wider economic impacts of extracting more revenue from the tobacco industry or tobacco trade?
- Who should administer fundraising? An existing system or organisation? A newly-formed organisation?
- How might we determine what is reasonable and affordable for tobacco companies to contribute?
    - How about the profits they make, relative to other commodities?

For peer review only - http://bmjopen.bmj.com/site/about/guidelines.xhtml

        - Prompt: the tobacco industry are one of the most profitable businesses in the world and make over £1bn in profit in the UK per year. Tobacco businesses tend to enjoy profit margins of up to 68%, compared to 15-20% in most staple consumer industries.
        - Prompt: however, tobacco sales have declined with the covid-19 pandemic
    - How about the amounts that they used to spend on advertising, before tobacco advertising was prohibited?
        - Prompt: evidence suggests that they used to spend £144m a year on advertising in the UK, adjusted for inflation
    - How about setting a target amount to raise in order to fund effective tobacco control activities at local, regional and national level, and then apportioning that across the tobacco industry?
    - How to apportion contributions between different companies?
        - Just the big four transnational tobacco companies, or any manufacturer or importer of tobacco operating in the UK?
        - Apportion by each company's share of the combustible tobacco product market? Or other products? Historical data vs current data?
- Should the tobacco industry be incentivised to move out of the combustible tobacco market, and encourage smokers to transition to alternative nicotine containing products?

<u>Part 3: How to disburse funds?</u> [Note: can focus on this more or less depending on interviewee's areas of expertise]

- What are the pros and cons of ring-fencing raised funds for a specific purpose vs. adding those funds to general public revenue?
- What should funds be used for?
    - Tobacco control measures
        - Cessation services
        - Preventing young people from initiating smoking
        - Fighting illicit tobacco
        - Mass media, social marketing and educational campaigns
        - Enforcement for age of sale compliance
        - Environment, parks and recreational resources
        - Any other ideas?
        - Is addition funding for tobacco control necessary if we are already trending towards tobacco being effectively obsolete?
    - General health costs or health promotion (not limited to tobacco)
    - Should the uses of funds be determined centrally or locally (i.e., by each devolved administration, or by regions/authorities within the devolved nations?)
    - Should the use of funds be set in stone, or flexible? How to make sure changes in the use of funds are sensible?
    - Is there a way to make sure funding addresses health inequalities?
- How to ensure that funds are disbursed for the intended purposes?
- Who should oversee and regulate the disbursement of funds, and why?
    - DHSC and their equivalents in the devolved nations?
    - A newly-formed semi-independent body?
    - A committee of appointed experts from government and civil society?

<u>Part 4: Advocacy, communication and legislation</u>

For peer review only - http://bmjopen.bmj.com/site/about/guidelines.xhtml

- How are the tobacco industry likely to respond to the announcement of a tax or levy?
  - Prompt: the industry has a track record of interfering with policy development and implementation
  - How to respond to likely industry critiques?
    - Example: tax is unfair and regressive
    - Example: revenue will not be used effectively
    - Example: taxes will be passed on to consumers
    - Example: higher costs will encourage illicit trade
    - Example: will harm the economy and endanger jobs
    - Example: revenue will decrease as purchasing decreases
    - Example: we're already working to transition people to reduced risk products therefore no further regulation necessary (CSR arguments)
- What other challenges do you anticipate in advocating for a new tax or levy?
  - Prompt: lobbying, media campaign, CSR rhetoric, reduced risk products, legal challenges
- What opportunities are there for advocating for a new tax or levy?
  - Prompt: Evidence of public support for raising taxes to pay for health and tobacco control
  - Prompt: Support from All Party group on Smoking and Health and the Smokefree Action Coalition
  - Prompt: money invested in tobacco control tends to create a large return on investment through healthcare savings

    Prompt: WHO FCTC requires that the government stringently regulates the tobacco market
- Is 'polluter pays' a useful way of framing a tobacco industry levy to fund tobacco control?
- What type of legislation is likely to be necessary to implement the types of measures we have discussed?
  - How long could we expect it to take

<u>Part 5: Contextual factors</u>

- How do you think the devolved nature of the UK will affect a new tax or levy?
  - How about giving devolved nations the option to opt into a scheme originating in Westminster? If so, to what extent should they have autonomy over how funds are used
  - How to calculate the distribution of funds between each of the devolved nations involved in the scheme?
- Do you have a feeling for how Brexit might affect a new tax or levy?
  - Are any challenges or opportunities presented by the change in the legal context caused by leaving the EU?
- How might Covid-19 affect a new tax or levy?
  - Prompt: tobacco sales have dropped during the pandemic
  - Prompt: covid-19 is expected to cause a global recession, how might that influence things?
  - Prompt: Would a tobacco control levy be an acceptable way of raising funds for economic recovery?
  - Prompt: Will Covid-19 raise the profile of NCD prevention measures such as tobacco control?
  - Prompt: Might the pandemic affect public and political attitudes to NHS funding? How might it change how we view policy solutions to NCDs and unhealthy commodities?
  - Prompt: How can we keep the issue of tobacco control on the political agenda?

For peer review only - http://bmjopen.bmj.com/site/about/guidelines.xhtml

Close interview

- Is there anything else you would like to add, that we haven't already talked about?
- Thank you very much for taking part.
- If appropriate – ask if they are interested in taking part in the second phase discussion groups.

For peer review only

For peer review only - http://bmjopen.bmj.com/site/about/guidelines.xhtml

Standards for Reporting Qualitative Research (SRQR)*

http://www.equator-network.org/reporting-guidelines/srqr/

Title and abstract

	Page/line no(s).
**Title** - Concise description of the nature and topic of the study Identifying the study as qualitative or indicating the approach (e.g., ethnography, grounded theory) or data collection methods (e.g., interview, focus group) is recommended	1
**Abstract** - Summary of key elements of the study using the abstract format of the intended publication; typically includes background, purpose, methods, results, and conclusions	2

Introduction

**Problem formulation** - Description and significance of the problem/phenomenon studied; review of relevant theory and empirical work; problem statement	4
**Purpose or research question** - Purpose of the study and specific objectives or questions	5

Methods

**Qualitative approach and research paradigm** - Qualitative approach (e.g., ethnography, grounded theory, case study, phenomenology, narrative research) and guiding theory if appropriate; identifying the research paradigm (e.g., postpositivist, constructivist/ interpretivist) is also recommended; rationale**	5 and 6
**Researcher characteristics and reflexivity** - Researchers' characteristics that may influence the research, including personal attributes, qualifications/experience, relationship with participants, assumptions, and/or presuppositions; potential or actual interaction between researchers' characteristics and the research questions, approach, methods, results, and/or transferability	14
**Context** - Setting/site and salient contextual factors; rationale**	5 and 6
**Sampling strategy** - How and why research participants, documents, or events were selected; criteria for deciding when no further sampling was necessary (e.g., sampling saturation); rationale**	5 and 6
**Ethical issues pertaining to human subjects** - Documentation of approval by an appropriate ethics review board and participant consent, or explanation for lack thereof; other confidentiality and data security issues	7
**Data collection methods** - Types of data collected; details of data collection procedures including (as appropriate) start and stop dates of data collection and analysis, iterative process, triangulation of sources/methods, and modification of procedures in response to evolving study findings; rationale**	5 and 6
**Data collection instruments and technologies** - Description of instruments (e.g., interview guides, questionnaires) and devices (e.g., audio recorders) used for data collection; if/how the instrument(s) changed over the course of the study	5 and 6
**Units of study** - Number and relevant characteristics of participants, documents, or events included in the study; level of participation (could be reported in results)	5 and 6

For peer review only - http://bmjopen.bmj.com/site/about/guidelines.xhtml

For peer review only

**Data processing** - Methods for processing data prior to and during analysis, including transcription, data entry, data management and security, verification of data integrity, data coding, and anonymization/de-identification of excerpts	6
**Data analysis** - Process by which inferences, themes, etc., were identified and developed, including the researchers involved in data analysis; usually references a specific paradigm or approach; rationale**	6
**Techniques to enhance trustworthiness** - Techniques to enhance trustworthiness and credibility of data analysis (e.g., member checking, audit trail, triangulation); rationale**	6

Results/findings

**Synthesis and interpretation** - Main findings (e.g., interpretations, inferences, and themes); might include development of a theory or model, or integration with prior research or theory	8-13
**Links to empirical data** - Evidence (e.g., quotes, field notes, text excerpts, photographs) to substantiate analytic findings	8-13

Discussion

**Integration with prior work, implications, transferability, and contribution(s) to the field -** Short summary of main findings; explanation of how findings and conclusions connect to, support, elaborate on, or challenge conclusions of earlier scholarship; discussion of scope of application/generalizability; identification of unique contribution(s) to scholarship in a discipline or field	14
**Limitations** - Trustworthiness and limitations of findings	16

Other

**Conflicts of interest** - Potential sources of influence or perceived influence on study conduct and conclusions; how these were managed	17
**Funding** - Sources of funding and other support; role of funders in data collection, interpretation, and reporting	17

*The authors created the SRQR by searching the literature to identify guidelines, reporting standards, and critical appraisal criteria for qualitative research; reviewing the reference lists of retrieved sources; and contacting experts to gain feedback. The SRQR aims to improve the transparency of all aspects of qualitative research by providing clear standards for reporting qualitative research.

**The rationale should briefly discuss the justification for choosing that theory, approach, method, or technique rather than other options available, the assumptions and limitations implicit in those choices, and how those choices influence study conclusions and transferability. As appropriate, the rationale for several items might be discussed together.

Reference:
O'Brien BC, Harris IB, Beckman TJ, Reed DA, Cook DA. **Standards for reporting qualitative research: a synthesis of recommendations.** *Academic Medicine*, Vol. 89, No. 9 / Sept 2014 DOI: 10.1097/ACM.0000000000000388

For peer review only - http://bmjopen.bmj.com/site/about/guidelines.xhtml

BMJ Open

Experts' views on how to design a Tobacco Control Fund in the UK

Journal:	*BMJ Open*
Manuscript ID	bmjopen-2022-066224.R2
Article Type:	Original research
Date Submitted by the Author:	15-Nov-2022
Complete List of Authors:	Hilton , Shona; University of Glasgow MRC/CSO Social and Public Health Sciences Unit<br>Smith, Marissa; University of Glasgow MRC/CSO Social and Public Health Sciences Unit<br>Buckton, Christina; University of Glasgow MRC/CSO Social and Public Health Sciences Unit<br>Patterson, Chris; University of Glasgow MRC/CSO Social and Public Health Sciences Unit
**Primary Subject Heading**:	Public health
Secondary Subject Heading:	Health policy, Health services research, Smoking and tobacco
Keywords:	PUBLIC HEALTH, QUALITATIVE RESEARCH, Health policy < HEALTH SERVICES ADMINISTRATION & MANAGEMENT

SCHOLARONE™
Manuscripts

I, the Submitting Author has the right to grant and does grant on behalf of all authors of the Work (as defined in the below author licence), an exclusive licence and/or a non-exclusive licence for contributions from authors who are: i) UK Crown employees; ii) where BMJ has agreed a CC-BY licence shall apply, and/or iii) in accordance with the terms applicable for US Federal Government officers or employees acting as part of their official duties; on a worldwide, perpetual, irrevocable, royalty-free basis to BMJ Publishing Group Ltd ("BMJ") its licensees and where the relevant Journal is co-owned by BMJ to the co-owners of the Journal, to publish the Work in this journal and any other BMJ products and to exploit all rights, as set out in our licence.

The Submitting Author accepts and understands that any supply made under these terms is made by BMJ to the Submitting Author unless you are acting as an employee on behalf of your employer or a postgraduate student of an affiliated institution which is paying any applicable article publishing charge ("APC") for Open Access articles. Where the Submitting Author wishes to make the Work available on an Open Access basis (and intends to pay the relevant APC), the terms of reuse of such Open Access shall be governed by a Creative Commons licence – details of these licences and which Creative Commons licence will apply to this Work are set out in our licence referred to above.

Other than as permitted in any relevant BMJ Author's Self Archiving Policies, I confirm this Work has not been accepted for publication elsewhere, is not being considered for publication elsewhere and does not duplicate material already published. I confirm all authors consent to publication of this Work and authorise the granting of this licence.

For peer review only

Experts' views on how to design a Tobacco Control Fund in the UK

Shona Hilton[1], Marissa J. Smith[1*], Christina Buckton[1], and Chris Patterson[1]

[1] MRC/CSO Social and Public Health Sciences Unit, University of Glasgow, Glasgow, UK

*Corresponding author
Email: Marissa.Smith@glasgow.ac.uk Tel: (+44) 0141 353 7500

Keywords: Public policy, Tobacco industry, Health services

Word count: 3500

For peer review only - http://bmjopen.bmj.com/site/about/guidelines.xhtml

Abstract

Objective

To explore expert views on the potential value, and approaches to establishing and administering a tobacco control fund in the United Kingdom (UK).

Design

Semi-structured interviews and follow-up discussion groups.

Subjects

Twenty-four UK and international experts on tobacco control regulation, public health, economics, or law from the academic, public, private and third sector.

Methods

Participants considered the relative merit of 1) general excise tax on retail tobacco sales; 2) ring-fenced hypothecation of excise taxes on retail tobacco sales; and 3) a direct levy on tobacco manufacturers. Preliminary synthesis of interview findings was deliberated upon in two follow-up discussion groups to identify key considerations for policy design.

Result

Most experts agreed that a ring-fenced tobacco control fund would be a valuable method of raising predictable and reliable funds from tobacco producers either using either companies' sales volume or market share as a way to establish the proportion they should pay. Experts predominantly recommended that a fund in the UK should be administered by a government body with devolved nation input and with an independent advisory group. They typically indicated that funding should be allocated yearly with a distribution at local, regional, and national levels to support smoking prevention and cessation rather than treatment activities with priority given to measures that tackle smoking-related inequalities.

Conclusion

For peer review only - http://bmjopen.bmj.com/site/about/guidelines.xhtml

There was overwhelming agreement by experts on the need to establish a tobacco control fund to help meet the proposed government tobacco-free targets to reduce adult smoking prevalence to 5% by 2030 (England) and 2034 (Scotland).

Strengths and limitations of this study

- Methodology includes semi-structured interviews with 24 UK and international experts on tobacco control regulation, public health, economics, or law from the academic, public, private and third sector, facilitating understandings of the potential value of a tobacco control fund in the UK.
- Follow-up discussion groups created informed dialogue between experts to collaboratively identify key considerations for policy design in this area by bringing together groups of policy actors diverse in terms of their specific areas of expertise and the sectors within which they have professional experience.
- Quantitative thematic analysis of the data allows depth of opinions but cannot offer predictions about the frequency of specific opinions with a wider population.
- The policy research offered new insights into an under-research area, but the complexity of policies and policymaking environments is such that transferring learning from one policy to a different policy is challenging.

For peer review only - http://bmjopen.bmj.com/site/about/guidelines.xhtml

Introduction

Worldwide tobacco kills more than 8 million people per annum [1] including nearly 100,000 preventable deaths in the UK [2]. Tobacco is highly addictive, there is no safe level of exposure and all forms of tobacco are harmful to health increasing the risk of cancers, heart disease, and other NCDs [1]. Despite a broad range of effective tobacco control policies, the tobacco trade continues to be highly profitable [3]. In contrast, the economic costs of tobacco use in society are greater than the costs for treating tobacco-related diseases. For example in the UK, revenue from excise duty on tobacco sales continues to be substantially lower than the health costs of smoking [4]. While UK smoking prevalence has declined precipitously in response to tobacco control action [5, 6], the smoking inequality gap has grown [6] as smoking contributes to poverty by diverting household spending from basic needs such as food and shelter to tobacco.

To further reduce smoking, the Westminster and Scottish governments have proposed targets to reduce adult smoking prevalence to 5% by 2030 and 2034, respectively [7, 8]. However, for these proposed targets to be met prevalence rates need to decline at a much faster rate [9] which may require additional tobacco control measures.

One policy option that has been proposed is to establish a ring-fenced tobacco control fund. This system for health promotion has been pursued in other countries including Australia, Vietnam, Korea, and Thailand [10, 11]. In the UK in 2015, Her Majesty's (HM) Treasury published their conclusions on an earlier consultation on the potential design of a levy on tobacco manufacturers and importers [12]. The consultation considered a tobacco levy under the administration of the existing corporation tax system, imposed on manufacturers and importers of products on which tobacco excise duty is paid [13]. Whilst the proposal received the support of a broad range of health charities, professional bodies, and academics; the UK Government decided not to pursue the tobacco levy, citing concerns that costs would be passed on to consumers and that tobacco sales are already subject to escalating duties [13]. Since the government rejected this tobacco industry levy, other fiscal approaches to tackling the harms caused by unhealthy products have been introduced, including Scotland's Minimum Unit Pricing for alcohol [14], and the 'soft hypothecation' of the UK's Soft Drinks Industry

For peer review only - http://bmjopen.bmj.com/site/about/guidelines.xhtml

Levy (SDIL) [15]. In light of an increasing political willingness to implement other fiscal interventions, and the continued advocacy for a tobacco control fund from the public health community [16, 17], raising revenue for a tobacco control fund from the tobacco industry remains a viable policy option. This paper explores contemporary views of UK and international tobacco control and public health experts on the potential value of, and approaches to establishing and administering a tobacco control fund. In doing so we identify key considerations for its design.

Methods

Interviews

We developed a purposive sampling frame to target UK and international experts in tobacco control regulation, public health, economics, or law from the academic, public, private and third sector. Twenty-four experts agreed to participate after reading the participant information sheet, privacy notice and signing the consent form. Eighteen were based in the UK, four in the United States, and two in South Africa. Table 1 illustrates the distribution of participants by the sector in which they primarily worked and their principal topic of work. Participants classified as 'public health' were those who work and publish in the broader area of public health. Participants classified as 'tobacco control' were those who had expertise in the area of tobacco control and predominately work and publish in the tobacco control area.

A semi-structured interview schedule (Appendix A) was informed by reviewing international academic and grey literature on tobacco control funds. The interviews were conducted between September 2020 and January 2021 by CP and CB. One interview was conducted by telephone and the remaining 23 interviews were conducted using Microsoft Teams video meetings. The interviews lasted between approximately 45 and 60 minutes, all were recorded and transcribed verbatim.

For peer review only - http://bmjopen.bmj.com/site/about/guidelines.xhtml

For peer review only

Table 1: Sample composition by primary sector of work and primary area of expertise

**Primary sector**	**Professional disciplinary approach to tobacco control**			**Total**
	Economics / Law	Public Health	Other	
Academia	6	3	0	**9**
Public sector	0	4	3	**7**
Third sector	1	1	5	**7**
Private sector	1	0	0	**1**
**Total**	**8**	**8**	**8**	**24**

Discussion Groups

In March 2021, two follow-up online discussion groups were conducted by CB and CP with nine individuals using Microsoft Teams. Participants were selected for these follow-ups based on their sectorial expertise and to represent key disciplines. The first discussion group (n=5) included three third sector professionals with expertise spanning tobacco control and public health advocacy and two academic economists. The second discussion group (n=4) included two public sector professionals with roles in tobacco control and public health policy and two academics with expertise in law and public health. The aim of these groups was to consider the synthesis of views from the interviews on the potential value of a tobacco control fund and to identify key considerations for policy design. Each discussion group lasted two hours, and group discussions were recorded for later checking against the minutes.

Analysis

For peer review only - http://bmjopen.bmj.com/site/about/guidelines.xhtml

For peer review only

We conducted thematic analysis of the data form the interview transcripts and discussion group minutes. The process followed Braun and Clarke's [18] six-phase framework for thematic analysis. The research team read and re-read the transcripts to become familiar with the data, and then iteratively constructed a coding frame to enable consistent organisation of relevant data. NVivo was used to organise categories on the basis of inductive themes that emerged from close reading of the, capture of both areas of agreement and less typical perspectives across a range of categories. The discussion group recordings and minutes were cross-compared with the interview coding frame to confirm and expand on codes relating to recommendations for policy design of a tobacco control fund. Where appropriate, the number of participants that gave specific opinions are presented as counts and proportions to help illustrate the balance of opinion with the sample. However, it must be noted that, given the qualitative methodology used in this study, these numbers cannot necessarily be generalised to any wider population.

Ethical approval for this study was granted by the College of Social Sciences Research Ethics Committee at the University of Glasgow (reference 400190213).

Patient and Public Involvement

None.

Results

The results are presented in accordance with the inductive coding categories developed during the analysis stage.

What is the potential value of a tobacco control fund?

For peer review only

There was general agreement that a tobacco control fund could be a valuable revenue for raising predictable and reliable funds direct from the tobacco industry. Typically, this was viewed as a way to boost current public health efforts:

> *"The more money that we can earmark, ring-fence into public health and tobacco control efforts the better from a public health point of view." (P02, academic, law)*

However, two participants cautioned that whilst such a fund was largely welcomed, it would be important that a tobacco control fund did not act as a disincentive for government funding or cutbacks to existing tobacco control activities. Participants also welcomed the fact that an industry-funded payment, would help hold the tobacco industry more accountable for the damage they cause to society, with one participant stating: *"There's some sort of nice symmetry about money from the tobacco industry being used to improve or solve some of the problems it creates" (P05, third sector, public health).* However, participants also warned that the funding mechanism of extracting money from the tobacco industry would need careful consideration so that the levy was not passed on to nicotine-dependent and socially deprived smokers, with one participant warning that:

> *"It doesn't really make sense, I think, to pursue further interventions that actually further widen the health inequality that we have." (P07, academic, public health)*

How might a tobacco control fund be designed?

Participants considered in more detail how a tobacco control fund might be designed to raise funds: 1) general excise tax on retail tobacco sales; 2) ring-fenced hypothecation of excise taxes on retail tobacco sales; and 3) a direct levy on tobacco manufacturers. Participants were asked to consider the relative merits of each funding approach.

General excise tax on retail tobacco sales

For peer review only

For peer review only - http://bmjopen.bmj.com/site/about/guidelines.xhtml

Participants were widely supportive of excise taxes, predominantly for their role in decreasing consumption, but also for their role in fundraising with some participants drawing on excise tax in Australia and New Zealand as useful models for the UK policymakers to consider. Participants highlighted the simplicity, efficiency, and political acceptability of excise tax as positive attributes of this approach. Some participants expressed doubt about the usefulness of excise taxes to fundraising given falling revenue with one academic stating:

> *"The UK I know is now sort of sitting at the top of that revenue situation where they increased excise tax, revenues are not increasing all that much because the excise taxes are very high already." (P01, academic, economics)*

In contrast, other participants suggested that the government can effectively control the extent to which taxes are passed on to consumers by capping retail prices, meaning that increasing specific excise tax can raise revenue while ensuring that retail prices do not increase so consumers do not bear the additional cost [19].

Ring-fenced hypothecated excise tax

Participants discussed the potential for some or all of excise taxation on retail tobacco sales to be hypothecated, meaning that it would be diverted into a specific fund instead of general government funds. This approach was viewed by participants as publicly acceptable as explained:

> *"Dealing with the consequences or addressing the harms that arise from the product I think is actually instinctively appealing to people." (P22, public sector, public health)*

However, while appealing in principle, participants overwhelmingly indicated that hypothecation would meet with too much opposition from HM Treasury, as noted by one academic:

> *"Politicians in general don't like it, they're very particular about being elected to do the right thing, and they wish to retain their independence and their freedom*

For peer review only - http://bmjopen.bmj.com/site/about/guidelines.xhtml

> *for manoeuvre. So, it can be a challenging negotiation that one." (P03, academic, public health)*

Despite this view, participants identified the UK's SDIL as a possible route to hypothecation but noted that the funds raised by SDIL were not ultimately ring-fenced for the purposes they originally presented to the public, with one participant noting:

> *"The sugar tax was pushed through with major public support on the basis of hypothecation. And then guess what? There was a crisis and the money, the revenues raised for the sugar tax miraculously didn't get spent on children's breakfast clubs and school sports but have been used to fill gaps in broader public health, and possibly NHS budgets as well. That's always a risk". (P03, academic, public health)*

This led to discussions about how to win political support for hypothecation and the merits of creating a general health fund instead of a tobacco control fund. As explained by one participant:

> *"I absolutely expect that it would be easier to convince policymakers, who generally don't like hypothecated taxes, [of the merits of a general health fund] so the more freedom that you give them, the more acceptable it's likely to be. But I would rather think it probably would be less acceptable to the public, because … if you're using the sort of, polluter pays type principle, then, you know, people expect that there is a direct consequence between those two things." (P22, public sector, public health)*

Another potential route to hypothecation discussed that bypassed HM Treasury was:

> *"If it was seen as a user fee done by the Department of Health and Social Care, then it would bypass the treasury's normal functioning." (P09, academic, economics)*

For peer review only

Bypassing HM Treasury was advocated by three participants who suggested taking inspiration from the UK's Pharmaceutical Price Regulation Scheme (PPRS) [20, 21], through which the Department for Health and Social Care (DHSC) (and not the treasury) receives excess profits from participating pharmaceutical companies and uses that funding to address shortfalls in NHS budgets due to expenditure on novel treatments. While this scheme is not an example of hypothecated tax, it was presented as a precedent for the DHSC receiving funds from industry, and an illustration of relevant administrative expertise within the DHSC, as explained:

> *"The important thing from the tobacco point of view is, you've established this principle of soft hypothecation where the rebates from the industry go back specifically for or back to the [DHSC], rather just the Treasury who just grab it." (P24, private sector, pharmaceuticals)*

A levy on the tobacco industry

Participants who favoured this approach (n=22, 92%) typically viewed it as a means to extract funds from industry instead of from consumers, which may be more appealing to the public and could help convince policymakers:

> *"I think politically it's more sellable to the public [than excise tax increases]." (P08, third sector, tobacco control)*

> *"I think that would be a decision-making factor for any governmental policy measure that got put forward, that it would be very much clear that the industry would be the contributor, not the public, if you like." (P22, public sector, public health)*

Controlling retail prices was deemed an essential part of ensuring that the cost of a levy is borne by the industry.

> *"The way that the tobacco companies are monopolies and making excess profits is*

For peer review only

> *because they are using gradual escalator duty increases to increase their own prices. So, you need to cap prices" (P24, private sector, pharmaceuticals).*

Conversely, some participants argued that retail prices should not be limited as price increases are beneficial in reducing consumption: As explained:

> *"When you do see tax increases, you tend to see over-shifting of the tax and using that as an opportunity to raise price and capitalise on at least the addicted consumers that are still in the market. So that is happening, but I don't know that that's necessarily a bad thing, because in the end those price increases are also very effective and leading to additional cessation and particularly in terms of preventing initiation." (P04, academic, economics)*

The PPRS was presented as a potential model for extracting industry profits outside of excise taxes, and one that has been refined over many years to limit potential loopholes. However, PPRS was generally considered to be of limited use in having real-world transferability from pharmaceuticals to tobacco. One public sector participant stated:

> *"The UK pharmaceutical market's status as a virtual monopsony differs starkly from the tobacco industry and suggested that such a scheme may incentivise the lowering of tobacco prices." (P14, public sector, tobacco control)*

In thinking about general principles of where the tobacco control fund might come from, participants discussed considered three options from 'profits', 'sales volume', or 'market share'. The option of a payment coming directly from 'profits' was largely discounted on the grounds that:

> *"Multinational companies are very good at moving money around and shifting profits to other countries with lower tax systems." (P03, academic, public health)*

The option of using 'sales volume' or 'market share' were both more popular as they were deemed more difficult for companies to obscure and shift money. Examples given were: "*The harm is linked*

For peer review only - http://bmjopen.bmj.com/site/about/guidelines.xhtml

For peer review only

to the sales volume of the product, not to the profits they make from it" (P23, third sector, tobacco control). Or that: "*Market share is the easiest way to do it. And you may want to average market share over the past 30 years or something like that to try to figure out what it is" (P02, academic, law).*

Other policy design considerations

After considering the different options for designing a tobacco control fund, participants considered other factors that would be essential for gaining public and political support. A key factor identified was the need for the fund to be administered by a government body with an independent advisory group to ensure transparent decision-making. As highlighted by one participant a requirement would be:

> *"A transparent body that both industry and [academic] researchers and the government had trust in to operate transparently and fairly and not be unduly influenced by any stakeholders, you just need to make it an independent body." (P05, third sector, economist)*

It was also agreed that the fund should be allocated yearly with distribution at local, regional, and national levels to support smoking prevention and cessation rather than treatment activities with priority given to tackling smoking-related inequalities in the most deprived areas. This was deemed important for:

> *"Making smoking obsolete, to massively benefit the most deprived communities both economically as well as in health terms." (P18, public sector, public health)*

Discussion

For peer review only - http://bmjopen.bmj.com/site/about/guidelines.xhtml

Experts considered three broad approaches to raising funds: raising existing excise tax on tobacco sales, introducing a hypothecated excise tax, and a tobacco industry levy. Each approach was assessed as having strengths and weaknesses, for example, raising excise taxes was seen as politically feasible and administratively simple, while hypothecation was seen as least politically plausible due to potential Treasury resistance and a tobacco levy was deemed as a logical advocacy route following the polluter pays principle to ensure the industry pays for its damage to society. Experts agreed that whichever mechanism is chosen, must be clearly guided by what the fund is directly trying to achieve. This is consistent with a recent Public Health England report on fiscal and pricing policies [22], which highlights that policy success depends on the clarity of policy goals. Most experts agreed that key principles underlying the design of a fund would be to collect predictable and reliable funds from transnational tobacco producers either from companies' sales volume or their market share as a way to assign responsibility and establish the proportion they should pay. There was agreement that any fundraising mechanism which extracts funds from industry and avoids the potentially regressive effects of price increases on consumers may be the optimal fundraising approach. However, there was acknowledgement that policy goals have trade-offs. For example to achieve both health promotion and revenue-raising objectives is possible within the same policy when demand for a product is relatively price-inelastic, as is the case with tobacco [22]. From this perspective, permitting costs to be passed on to customers and ensuring that costs are paid by industry may each be valid goals, and designing the policy requires skill. The implementation of other fiscal interventions to tackle the harms caused by unhealthy products, such as the SDIL, our research has shown the political willingness to establish a tobacco control fund. Experts described the potential for resistance from the Treasury, politicians and the public to these three potential tobacco control fund proposals. Industry resistance and influence is relevant in terms of both policy acceptability and ensuring compliance with World Health Organisation (WHO) Framework Convention Tobacco Control (FCTC) [23], thus future research could explore the potential for resistance from industry actors concerning a the design of a tobacco control fund in the UK.

For peer review only - http://bmjopen.bmj.com/site/about/guidelines.xhtml

In considering the policy approaches to raise funds lessons may be learned from other countries such as Australia, Thailand, Vietnam, and Korea who have implemented this system for health promotion [10, 11, 24]. Australia have been leaders in establishing and administering tobacco control funding. The Victorian Health Promotion Foundation (VicHealth), established in 1987, was the first foundation to be funded by a tax on tobacco with a legislative mandate to promote health in the state of Victoria, Australia [25, 26]. The levy was set at 5% and this increased the state tobacco licence fee from 25% to 30%. [27] In the first year, the money raised approximately $23 million and this was paid directly into the foundation [27]. It was regarded an inspiration [25, 28] and subsequently, led to the establishment of the West Australian Health Promotion Foundation (Healthway) [25]. Relevant here is that VicHealth is a self-governing statutory board enabling it to be an independent board. This independence allows the Foundation to distance themselves from tobacco industry influence.

Similar to VicHealth, the Thai Health Promotion Foundation (ThaiHealth) is a self-governing statutory board, funded by industry money but independent from tobacco industry interference [25]. Revenue for ThaiHealth was established from a new 2% earmarked tax on tobacco and alcohol importers and manufacturers to support tobacco control and health promotion efforts [29]. Vietnam and Korea have also adopted similar funding models [30] mobilising financial resources to strengthen cessation services and develop interventions to help tobacco growers change their occupations [25, 31, 32].

In the UK context, there was good agreement that the fund should be focused on tackling smoking-related health inequalities and preventing people from starting to smoke and helping them to quit rather than treating smoking-related diseases. Experts in this current study also suggested that the fund should be ring-fenced and allocated yearly with distribution at local, regional, and national levels to support a comprehensive tobacco control plan towards meeting government targets. This is similar to the way VicHealth operate where their goals are aligned with government targets for example the 10-year goal that 400,000 more Victorians would be tobacco-free by 2023 [33]. Experts in this study also identified that the fund should be run in an independent and transparent way without any interference or input from the tobacco industry as VicHealth and ThaiHealth have done.

For peer review only - http://bmjopen.bmj.com/site/about/guidelines.xhtml

Several limitations in this study are worth noting. The qualitative nature of data offers depth of opinion within the research sample but does not offer any predictions about the frequency of specific stances within any wider population. We were satisfied that the diversity of professional experience and expertise across these 24 participants provided us with a sample that represented the breadth of perspectives likely to be found within our target population. The value of qualitative policy research is in identifying useful reasoning and novel ideas, not making generalisations about how commonplace specific opinions are. This study was also affected by certain limitations inherent to policy research. The complexity of policies and policymaking environments is such that transferring learning from one policy to a different policy is challenging [34]. For example, the US tobacco Master Settlement Agreement (MSA) may contain valuable lessons for tobacco control policy in the UK, but the importance of the differences in time periods and legislative contexts cannot be discounted. As such, few participants possessed the breadth of context and knowledge to be able to present comprehensive recommendations for policy. More commonly, participants presented in-depth knowledge in specific areas or general principles for policymaking. However, this study offers new insights into an under-researched area. While the interviews were valuable in producing rich individual accounts into relevant aspects of tobacco control, the key benefit of the discussion groups was in creating an informed dialogue between experts. Together this data offered a valuable means of arriving at grounded policy recommendations through interdisciplinary discussion, useful in policy research due to the extent to which policy is constructed through the discursive engagement of different coalitions of policy actors [35]. Another strength was using online data collection which proved to be straightforward reduced geographical barriers to participation among world-leading experts in the UK, the US, and South Africa.

Conclusion

Smoking remains a leading preventable cause of death and disease in the UK with much of this impacting the poorest communities. The implementation of a tobacco control fund would help meet the proposed government tobacco-free targets. However, there is no 'one size fits all' template for

For peer review only - http://bmjopen.bmj.com/site/about/guidelines.xhtml

such fund, the structure, and operations of the fund would need to adapt to other countries to fit the culture, government ideology, and social context. This research shows that experts support the introduction of a tobacco control fund to reduce inequalities in health and achieve the English and Scottish targets of reducing adult smoking prevalence to 5% by 2030 and 2034, respectively. e It provides early insights into how a fund might be established and administered in the UK and sets out key foundational principles that must be engaged with in designing a tobacco control fund policy in the UK. Importantly, although there was no one funding approach had unanimous support, experts agreed that establishing an 'imperfect policy' that provides dedicated funding is preferable to delay and inaction.

Contributions: **Shona Hilton:** Conceptualisation, Data Curation, Methodology, Validation, Writing-Original draft preparation. **Marissa J. Smith:** Data Curation, Visualisation**,** Writing - Review & Editing. **Christina Buckton:** Conceptualisation, Data Curation, Methodology, Investigation, Validation, Writing - Review & Editing. **Chris Patterson:** Conceptualisation, Data Curation, Methodology, Investigation, Validation, Writing - Review & Editing.

Competing interests: No conflicts of interest.

Funding: This work was supported by the Medical Research Council **(**MC_UU_00022/2 and (MC_UU_12017/15), Chief Scientist Office of the Scottish Government Health Directorates (SPHSU15 and SPHSU17) and Cancer Research UK Grant PPRCTAGPJT\100003.

Data sharing statement: All data relevant to the study are included in the article or uploaded as supplementary information.

Ethics Approval statement: Ethical approval for this study was granted by the College of Social Sciences Research Ethics Committee at the University of Glasgow (reference number: 400190213).

For peer review only

References

1. WHO. Tobacco: Leading cause of death, illness and impoverishment 2021 [Available from: https://www.who.int/en/news-room/fact-sheets/detail/tobacco.
2. ASH. Written evidence submitted by Action on Smoking and Health. 2020.
3. Branston JR, Gilmore A. The extreme profitability of the UK tobacco market and the rationale for a new tobacco levy. University of Bath; 2015.
4. Branston JR, Gilmore AB. The failure of the UK to tax adequately tobacco company profits. 2019;42(1):69-76.
5. Cancer Research UK. Tobacco statistics 2020 [Available from: https://www.cancerresearchuk.org/health-professional/cancer-statistics/risk/tobacco.
6. Office for National Statistics. Adult smoking habits in the UK: 2019. 2020.
7. HM Government. Advancing our health: prevention in the 2020s – consultation document 2019 [Available from: https://www.gov.uk/government/consultations/advancing-our-health-prevention-in-the-2020s/advancing-our-health-prevention-in-the-2020s-consultation-document.
8. The Scottish Government. Creating a Tobacco-Free Generation: A Tobacco Control Strategy for Scotland. Edinburgh: The Scottish Government; 2013.
9. Cancer Intelligence Team. Smoking prevalence projections for England, Scotland, Wales and Northern Ireland, based on data to 2018/19: Cancer Research UK; 2020 [
10. Nam EW, Hasegawa T, Davies JK, Ikeda N. Health promotion policies in the Republic of Korea and Japan: a comparative study. 2006;13(1):20-8.
11. Nguyen DT, Luong KN, Phan HT, Tran AT, Dao ST, Poudel AN, et al. Cost-Effectiveness of Population-Based Tobacco Control Interventions on the Health Burden of Cardiovascular Diseases in Vietnam. APJPH. 2021;33(8):854-60.
12. HM Treasury. Tobacco levy: response to the consultation. HM Treasury; 2015.
13. HM Treasury. Tobacco levy: consultation. HM Treasury; 2014.
14. Scottish Government. Minimum Unit Pricing 2017 [Available from: http://www.gov.scot/Topics/Health/Services/Alcohol/minimum-pricing.
15. HM Revenue & Customs. Policy paper: Soft Drinks Industry Levy 2016 [Available from: https://www.gov.uk/government/publications/soft-drinks-industry-levy/soft-drinks-industry-levy.
16. Cancer Research UK. Cancer Research UK urges government to make big tobacco cough up 2016 [Available from: https://www.cancerresearchuk.org/about-us/cancer-news/press-release/2016-01-26-cancer-research-uk-urges-government-to-make-big-tobacco-cough-up.
17. Action on Smoking and Health. Big Tobacco should pay for the damage it does 2018 [Available from: https://ash.org.uk/media-and-news/blog/big-tobacco-should-pay-for-the-damage-it-does/.
18. Braun V, Clarke V. Thematic analysis. APA handbook of research methods in psychology, Vol 2: Research designs: Quantitative, qualitative, neuropsychological, and biological. APA handbooks in psychology®. Washington, D.C., USA: American Psychological Association; 2012. p. 57-71.
19. Branston JR, Gilmore AB. The case for Ofsmoke: the potential for price cap regulation of tobacco to raise £500 million per year in the UK. Tob Control. 2014;23(1):45-50.
20. Collier J. The pharmaceutical price regulation scheme. BMJ. 2007;334(7591):435-6.
21. Rodwin MA. How the United Kingdom Controls Pharmaceutical Prices and Spending: Learning From Its Experience. 2021;51(2):229-37.
22. Public Health England. Fiscal and pricing policies: evidence report and framework. 2018.
23. World Health Organisation. The WHO framework convention on tobacco control: an overview. Geneva, Switzerland: World Health Organisation; 2015.
24. Smith KE, Savell E, Gilmore AB. What is known about tobacco industry efforts to influence tobacco tax? A systematic review of empirical studies. Tob Control. 2013;22(2):144-53.
25. International Union Against Tuberculosis and Lung Disease. Sustainable Funding Models for Tobacco Control: a Discussion Paper. 2014.
26. Bonito S. VicHealth: The World's First Health Promotion Foundation. Michigan: University of Michigan; 2014.
27. Victorian Health Promotion Foundation. The Story of VicHealth. Carlton, Australia; 2005.

For peer review only

For peer review only - http://bmjopen.bmj.com/site/about/guidelines.xhtml

28. Rechter J. Innovate, Inform, Integrate: VicHealth is transforming to meet innovation and the new health promotion challenges. 2015;30(3):413-7.
29. Hamann SL, Mock J, Hense S, Charoenca N, Kungskulniti N. Building tobacco control research in Thailand: meeting the need for innovative change in Asia. Health Res Policy Sys. 2012;10(1):3.
30. Decision on the establishment and approval of the regulation on the organization and operation of the Vietnam Tobacco Control Fund, 47/2013/QĐ-TTg (2013).
31. Law on Prevention and Control of Tobacco Harms, 09/2012/QH13 (2012).
32. National Tobacco Control Center. Tobacco taxation in Korea 2016 [Available from: https://nosmk.khealth.or.kr/ntcc/eng/subIndex/569.do.
33. VicHealth. VicHealth Tobacco Strategy 2019–2023 2019 [updated 25 February 2020. Available from: https://www.vichealth.vic.gov.au/media-and-resources/publications/vichealth-tobacco-strategy.
34. Cairney P. Complexity theory in political science and public policy. 2012;10(3):346-58.
35. Herzog C, Ali C. Elite interviewing in media and communications policy research. 2015;11(1):37-54.

For peer review only

For peer review only - http://bmjopen.bmj.com/site/about/guidelines.xhtml

Appendix A: Interview topic guide

Part 1: Introduction/background

- First of all, thank you for taking part in this research.
- I'm [researcher name], a research assistant at the University of Glasgow with an interest in the communication of health issues and policies.
- Your will be aware from the participant information sheet that this is a project funded by Cancer Research UK to collect expert views on the extent to which a direct levy on the tobacco industry (a so called 'polluter pays' levy) might be an effective tobacco control measure. We are looking to compare the different forms of a levy and their potential impacts; consider how funds generated could be used for tobacco control activities; learn from international case studies; and if there is support for the levy, to provide recommendations for the next steps in advocating for it. Cancer Research UK (CRUK) will use the outcome of this project to inform their future tobacco control policy strategy.
- We are interviewing a mix of stakeholders with relevant expertise including legal experts, economists, financial or tax advisors, industry experts or representative bodies, charities and harm-reduction groups and academics or researchers.

Key points for consent:

- Can I confirm that you have received the participant information sheet and signed and returned your consent form.
- Just to reiterate, your taking part is voluntary, and you are free to withdraw at any time without giving a reason, up until the point where the data is published in an NHS Health Scotland report.
- Your participation will be anonymous. Excerpts from the interview may be quoted verbatim in a report and a paper, but quotations will be anonymised to avoid accidentally disclosing your identity. We realise that within certain policy communities it may be possible for others to identify you from your experiences of specific policies, as such we will take care to anonymise quotations as appropriate to avoid accidental disclosure. Non-anonymised interview recordings and transcripts will be destroyed securely upon completion of this research, but anonymised transcripts and consent forms will be stored securely by the University of Glasgow for a period of 10 years for the purposes of ensuring research integrity.
- Finally, the study has been approved by the Ethics Committee of the College of Social Sciences at the University of Glasgow.

Verbal confirmation of consent:

- Do you consent to take part in this research, and do you give consent for me to record this interview?
- Can you describe your professional role, and how it relates to taxation, tobacco control or the regulation of unhealthy commodities?

<u>Part 2: How to raise funds?</u> [Note: can focus on this more or less depending on interviewee's areas of expertise]

- What different mechanisms are you aware of for raising funds from the tobacco industry?
    - How about hypothecated excise taxes? [explain if necessary]

For peer review only - http://bmjopen.bmj.com/site/about/guidelines.xhtml

          - Do you think excise taxes are an effective away to raise revenue?
            - What are the pros and cons?
            - Could that revenue be effectively ring-fenced for specific purposes?
          - What specifically should a tax be applied to?
            - Sales at retail
            - Sales between manufacturers and distributors, or between distributors and retailers?
            - Sales crossing international boundaries
          - [If participant is favourable towards excise taxes]
            - Should tax rates differ between different tobacco products, such as heat not burn products, or should there be a flat rate of tax across all tobacco products?
            - Should taxes be direct (fixed amount per unit) or ad valorem (percentage of price)?
        - How about an industry levy? [explain if necessary – mandatory direct tax charged to the industry]
          - Are you aware of any direct taxes or levies that already exist?
            - Prompt: Soft drinks industry levy
            - Prompt: Pharmaceutical Price Regulation Scheme (UK)
            - Prompt: Tobacco product user fees (US)
        - Are there any examples of where other countries have used taxes or levies to raise funds from the tobacco industry for tobacco control? What lessons might we take from these?
        - Are there any novel approaches that should be considered?
          - Are there useful lessons to be learned from other pricing and taxation schemes (ECO, MUP, SDIL, single-use bag tax, Scottish Landfill Communities Fund)
- How far in advance should a fundraising scheme be planned?
  - Should funding rise over time, remain steady or drop over time?
  - At what point will it no longer be necessary?
  - What time of ongoing surveillance of the market will be necessary?
- What ways might the tobacco industry try to avoid contributing more? What are the implications of these strategies? How might we prevent these?
  - Prompt: TTCs 'hiding' profits in other countries
  - Prompt: over-shifting (increase prices on top of tax increases)
  - Prompt: under-shifting (absorb tax increases to keep retail prices stable
  - Prompt: brand-shifting (over- and under-shift different brands to encourage continued use and initiation of tobacco
  - Prompt: Collusion between TTCs to keep prices low
  - Prompt: Counter-marketing to undermine investment in public communication campaigns
  - Prompt: Subversion of a tobacco control fund idea eg: PMI's Tobacco Transition Fund
  - Prompt: Use of a TTF to gain access to policy making
  - Anything else?
- Is raising the price of tobacco products desirable? How does it relate to inequalities?
  - [If undesirable] Is capping prices a good idea? Is it practically and legally feasible?
  - Can you think of any wider economic impacts of extracting more revenue from the tobacco industry or tobacco trade?
- Who should administer fundraising? An existing system or organisation? A newly-formed organisation?
- How might we determine what is reasonable and affordable for tobacco companies to contribute?
  - How about the profits they make, relative to other commodities?

For peer review only - http://bmjopen.bmj.com/site/about/guidelines.xhtml

    - Prompt: the tobacco industry are one of the most profitable businesses in the world and make over £1bn in profit in the UK per year. Tobacco businesses tend to enjoy profit margins of up to 68%, compared to 15-20% in most staple consumer industries.
    - Prompt: however, tobacco sales have declined with the covid-19 pandemic
  - How about the amounts that they used to spend on advertising, before tobacco advertising was prohibited?
    - Prompt: evidence suggests that they used to spend £144m a year on advertising in the UK, adjusted for inflation
  - How about setting a target amount to raise in order to fund effective tobacco control activities at local, regional and national level, and then apportioning that across the tobacco industry?
  - How to apportion contributions between different companies?
    - Just the big four transnational tobacco companies, or any manufacturer or importer of tobacco operating in the UK?
    - Apportion by each company's share of the combustible tobacco product market? Or other products? Historical data vs current data?
- Should the tobacco industry be incentivised to move out of the combustible tobacco market, and encourage smokers to transition to alternative nicotine containing products?

<u>Part 3: How to disburse funds?</u> [Note: can focus on this more or less depending on interviewee's areas of expertise]

- What are the pros and cons of ring-fencing raised funds for a specific purpose vs. adding those funds to general public revenue?
- What should funds be used for?
  - Tobacco control measures
    - Cessation services
    - Preventing young people from initiating smoking
    - Fighting illicit tobacco
    - Mass media, social marketing and educational campaigns
    - Enforcement for age of sale compliance
    - Environment, parks and recreational resources
    - Any other ideas?
    - Is addition funding for tobacco control necessary if we are already trending towards tobacco being effectively obsolete?
  - General health costs or health promotion (not limited to tobacco)
  - Should the uses of funds be determined centrally or locally (i.e., by each devolved administration, or by regions/authorities within the devolved nations?)
  - Should the use of funds be set in stone, or flexible? How to make sure changes in the use of funds are sensible?
  - Is there a way to make sure funding addresses health inequalities?
- How to ensure that funds are disbursed for the intended purposes?
- Who should oversee and regulate the disbursement of funds, and why?
  - DHSC and their equivalents in the devolved nations?
  - A newly-formed semi-independent body?
  - A committee of appointed experts from government and civil society?

<u>Part 4: Advocacy, communication and legislation</u>

For peer review only - http://bmjopen.bmj.com/site/about/guidelines.xhtml

- How are the tobacco industry likely to respond to the announcement of a tax or levy?
    - Prompt: the industry has a track record of interfering with policy development and implementation
    - How to respond to likely industry critiques?
        - Example: tax is unfair and regressive
        - Example: revenue will not be used effectively
        - Example: taxes will be passed on to consumers
        - Example: higher costs will encourage illicit trade
        - Example: will harm the economy and endanger jobs
        - Example: revenue will decrease as purchasing decreases
        - Example: we're already working to transition people to reduced risk products therefore no further regulation necessary (CSR arguments)
- What other challenges do you anticipate in advocating for a new tax or levy?
    - Prompt: lobbying, media campaign, CSR rhetoric, reduced risk products, legal challenges
- What opportunities are there for advocating for a new tax or levy?
    - Prompt: Evidence of public support for raising taxes to pay for health and tobacco control
    - Prompt: Support from All Party group on Smoking and Health and the Smokefree Action Coalition
    - Prompt: money invested in tobacco control tends to create a large return on investment through healthcare savings

      Prompt: WHO FCTC requires that the government stringently regulates the tobacco market
- Is 'polluter pays' a useful way of framing a tobacco industry levy to fund tobacco control?
- What type of legislation is likely to be necessary to implement the types of measures we have discussed?
    - How long could we expect it to take

Part 5: Contextual factors

- How do you think the devolved nature of the UK will affect a new tax or levy?
    - How about giving devolved nations the option to opt into a scheme originating in Westminster? If so, to what extent should they have autonomy over how funds are used
    - How to calculate the distribution of funds between each of the devolved nations involved in the scheme?
- Do you have a feeling for how Brexit might affect a new tax or levy?
    - Are any challenges or opportunities presented by the change in the legal context caused by leaving the EU?
- How might Covid-19 affect a new tax or levy?
    - Prompt: tobacco sales have dropped during the pandemic
    - Prompt: covid-19 is expected to cause a global recession, how might that influence things?
    - Prompt: Would a tobacco control levy be an acceptable way of raising funds for economic recovery?
    - Prompt: Will Covid-19 raise the profile of NCD prevention measures such as tobacco control?
    - Prompt: Might the pandemic affect public and political attitudes to NHS funding? How might it change how we view policy solutions to NCDs and unhealthy commodities?
    - Prompt: How can we keep the issue of tobacco control on the political agenda?

For peer review only - http://bmjopen.bmj.com/site/about/guidelines.xhtml

Close interview

- Is there anything else you would like to add, that we haven't already talked about?
- Thank you very much for taking part.
- If appropriate – ask if they are interested in taking part in the second phase discussion groups.

For peer review only

Standards for Reporting Qualitative Research (SRQR)*

http://www.equator-network.org/reporting-guidelines/srqr/

Page/line no(s).

Title and abstract

**Title** - Concise description of the nature and topic of the study Identifying the study as qualitative or indicating the approach (e.g., ethnography, grounded theory) or data collection methods (e.g., interview, focus group) is recommended	1
**Abstract** - Summary of key elements of the study using the abstract format of the intended publication; typically includes background, purpose, methods, results, and conclusions	2

Introduction

**Problem formulation** - Description and significance of the problem/phenomenon studied; review of relevant theory and empirical work; problem statement	3
**Purpose or research question** - Purpose of the study and specific objectives or questions	4

Methods

**Qualitative approach and research paradigm** - Qualitative approach (e.g., ethnography, grounded theory, case study, phenomenology, narrative research) and guiding theory if appropriate; identifying the research paradigm (e.g., postpositivist, constructivist/ interpretivist) is also recommended; rationale**	5 and 6
**Researcher characteristics and reflexivity** - Researchers' characteristics that may influence the research, including personal attributes, qualifications/experience, relationship with participants, assumptions, and/or presuppositions; potential or actual interaction between researchers' characteristics and the research questions, approach, methods, results, and/or transferability	14
**Context** - Setting/site and salient contextual factors; rationale**	5 and 6
**Sampling strategy** - How and why research participants, documents, or events were selected; criteria for deciding when no further sampling was necessary (e.g., sampling saturation); rationale**	5 and 6
**Ethical issues pertaining to human subjects** - Documentation of approval by an appropriate ethics review board and participant consent, or explanation for lack thereof; other confidentiality and data security issues	7
**Data collection methods** - Types of data collected; details of data collection procedures including (as appropriate) start and stop dates of data collection and analysis, iterative process, triangulation of sources/methods, and modification of procedures in response to evolving study findings; rationale**	5 and 6
**Data collection instruments and technologies** - Description of instruments (e.g., interview guides, questionnaires) and devices (e.g., audio recorders) used for data collection; if/how the instrument(s) changed over the course of the study	5 and 6
**Units of study** - Number and relevant characteristics of participants, documents, or events included in the study; level of participation (could be reported in results)	5 and 6

For peer review only - http://bmjopen.bmj.com/site/about/guidelines.xhtml

**Data processing** - Methods for processing data prior to and during analysis, including transcription, data entry, data management and security, verification of data integrity, data coding, and anonymization/de-identification of excerpts	6
**Data analysis** - Process by which inferences, themes, etc., were identified and developed, including the researchers involved in data analysis; usually references a specific paradigm or approach; rationale**	6
**Techniques to enhance trustworthiness** - Techniques to enhance trustworthiness and credibility of data analysis (e.g., member checking, audit trail, triangulation); rationale**	6

Results/findings

**Synthesis and interpretation** - Main findings (e.g., interpretations, inferences, and themes); might include development of a theory or model, or integration with prior research or theory	8-13
**Links to empirical data** - Evidence (e.g., quotes, field notes, text excerpts, photographs) to substantiate analytic findings	8-13

Discussion

**Integration with prior work, implications, transferability, and contribution(s) to the field** - Short summary of main findings; explanation of how findings and conclusions connect to, support, elaborate on, or challenge conclusions of earlier scholarship; discussion of scope of application/generalizability; identification of unique contribution(s) to scholarship in a discipline or field	14
**Limitations** - Trustworthiness and limitations of findings	16

Other

**Conflicts of interest** - Potential sources of influence or perceived influence on study conduct and conclusions; how these were managed	17
**Funding** - Sources of funding and other support; role of funders in data collection, interpretation, and reporting	17

*The authors created the SRQR by searching the literature to identify guidelines, reporting standards, and critical appraisal criteria for qualitative research; reviewing the reference lists of retrieved sources; and contacting experts to gain feedback. The SRQR aims to improve the transparency of all aspects of qualitative research by providing clear standards for reporting qualitative research.

**The rationale should briefly discuss the justification for choosing that theory, approach, method, or technique rather than other options available, the assumptions and limitations implicit in those choices, and how those choices influence study conclusions and transferability. As appropriate, the rationale for several items might be discussed together.

Reference:
O'Brien BC, Harris IB, Beckman TJ, Reed DA, Cook DA. **Standards for reporting qualitative research: a synthesis of recommendations.** *Academic Medicine*, Vol. 89, No. 9 / Sept 2014
DOI: 10.1097/ACM.0000000000000388

For peer review only - http://bmjopen.bmj.com/site/about/guidelines.xhtml

For peer review only

For peer review only - http://bmjopen.bmj.com/site/about/guidelines.xhtml
